# Cannabinoid exposure across substance use disorders: Short-term symptom benefits without sustained therapeutic gains in a tier-weighted systematic review

**DOI:** 10.1192/j.eurpsy.2026.12236

**Published:** 2026-07-17

**Authors:** David Zammit Dimech, Audrey-Ann Zammit Dimech, Louise Grech, Anthony Serracino Inglott

**Affiliations:** 1 Division of Clinical and Surgical Sciences, University of Edinburgh, Edinburgh, United Kingdom; 2 University of Malta Faculty of Medicine & Surgery, Malta

**Keywords:** cannabinoids, evidence synthesis, opioid use disorder, substance use disorders, systematic review

## Abstract

**Background:**

Cannabinoids are increasingly discussed as adjuncts in addiction treatment, yet whether they improve clinically meaningful substance use disorder (SUD) outcomes beyond short-term symptom relief is unresolved. We determined whether cannabinoid exposure confers directional efficacy across opioid, alcohol, cocaine, tobacco, and methamphetamine use disorders, distinguishing symptomatic targets from sustained therapeutic outcomes.

**Methods:**

PubMed and Embase (1975–2025) were searched for human studies evaluating cannabinoid exposure in relation to SUD outcomes. Two reviewers independently screened and extracted data. Risk of bias was assessed using RoB 2 for randomized controlled trials (RCTs), ROBINS-I for cohort studies, and JBI checklists for cross-sectional, case, and qualitative designs. Six prespecified endpoints (treatment retention, relapse, abstinence, craving, withdrawal severity, consumption) were mapped to each target SUD. Following Synthesis Without Meta-analysis (SWiM) guidance, structured narrative synthesis used a design-based weighting scheme (RCT 1.00 to qualitative 0.25). PROSPERO: CRD420251151193.

**Results:**

Ninety-seven studies (41,954 participants) contributed 195 endpoint instances: 89 Beneficial (45.6%), 80 No Significant Effect (41.0%), 12 Mixed/Partial (6.2%), and 14 Harmful/Inferior (7.2%). Short-term symptom targets accounted for most Beneficial findings (76.4%). Sustained outcomes were predominantly No Significant Effect, most pronounced in opioid use disorder. Beneficial symptom findings derived overwhelmingly from weaker study designs (craving 81.5%; withdrawal severity 85.7%; consumption 80.0%).

**Conclusions:**

Cannabinoids confer short-horizon symptomatic benefits but do not demonstrate efficacy for sustained abstinence, relapse prevention, or retention, most clearly in opioid use disorder, where evidence is strongest. Findings for other disorders remain preliminary. Adequately powered adjunctive randomized trials with biochemically verified endpoints are needed.

## Introduction

Substance use disorders (SUDs) remain a leading cause of preventable morbidity and mortality worldwide, with opioids [1, 2], alcohol [3, 4], cocaine [5, 6], and tobacco [7, 8] accounting for a substantial share of health loss and service utilization. Clinical interest in cannabinoids has risen rapidly alongside shifting policies and widespread real-world use [9, 10], yet evidence on whether cannabis or cannabinoid formulations improve SUD outcomes is mixed [11,12]. Trials and observational studies vary markedly in exposure definitions, comparators, and outcome windows [13], and many reports privilege short-horizon laboratory or symptom readouts that may not translate into durable clinical benefit [14, 15]. Outcomes across this literature are also ascertained in very different ways. Some are confirmed objectively, through biochemical testing or administrative records, while others rest on participant self-report, which is more vulnerable to recall, expectancy, and social desirability bias. Because a finding supported by objective verification carries more evidential weight than one resting on self-report alone, the method of ascertainment is itself relevant to how cannabinoid effects should be judged. At the same time, patients and addiction treatment services need pragmatic answers about whether cannabinoids reduce relapse or improve retention in treatment programs [16, 17], yet actionable, disorder-specific guidance remains limited [18].

Previous systematic reviews have either addressed cannabinoids within broad psychiatric or health syntheses where SUDs occupied a secondary role, represented by small subsets of studies heavily skewed toward cannabis use disorder (CUD), or focused on SUDs but with fragmented evidence bases constrained to single disorders or narrow populations. None has synthesized the full breadth of human evidence across the principal SUDs within a single design-weighted framework calibrated to distinguish symptomatic targets from sustained therapeutic outcomes. Each element of this gap matters for a different reason, and each may contribute to the apparent inconsistency of the literature. Breadth across disorders is important because the contexts, mechanisms, and treatment goals of opioid, alcohol, cocaine, tobacco, and methamphetamine use disorders differ, so a cannabinoid effect present in one disorder need not generalize to another. Reviews confined to single disorders or skewed toward CUD cannot reveal such variation, and reading their conclusions side by side can make coherent patterns at the disorder level appear as unexplained disagreement. The design-weighted element matters in a different way, because studies of unequal internal validity are otherwise treated as interchangeable, even though weaker designs are more exposed to confounding and selection effects that tend to favor positive signals. When beneficial results cluster in lower-tier designs and null results in stronger ones, an unweighted reading registers the field as mixed when the discrepancy in fact tracks evidential strength. The distinction between symptomatic and sustained outcomes is no less consequential because a treatment can ease short-term symptoms without altering the longer-term course of a disorder. Where these two classes of endpoint are pooled together, a benefit on craving or withdrawal sitting alongside an absent effect on retention or abstinence reads as contradiction rather than as a coherent divergence by outcome horizon. Meanwhile, adequately powered randomized controlled trials (RCTs) within individual SUDs remain too few to define clinical efficacy independently, and the heterogeneity of the evidence base makes conventional meta-analysis unsuitable. A cross-SUD synthesis that integrates diverse designs while weighting conclusions by evidential strength can therefore yield a more clinically informative picture than any prior review.

We, therefore, synthesized human studies encompassing opioid, alcohol, cocaine, tobacco, and methamphetamine use disorders to determine whether cannabis or specific cannabinoid formulations confer clinically meaningful benefit across SUDs. Studies were classified and interpreted according to design quality and internal validity, and results were organized by SUD endpoint so that stronger designs and sustained therapeutic outcomes informed the overall conclusions. This endpoint-centered, tiered structure preserves methodological transparency and enables inferences that inform clinical practice while acknowledging where mechanistic or intermediate signals point to plausible pathways that require confirmatory trials targeting sustained clinical outcomes.

## Methods

### Search strategy and selection criteria

**Figure 1.**
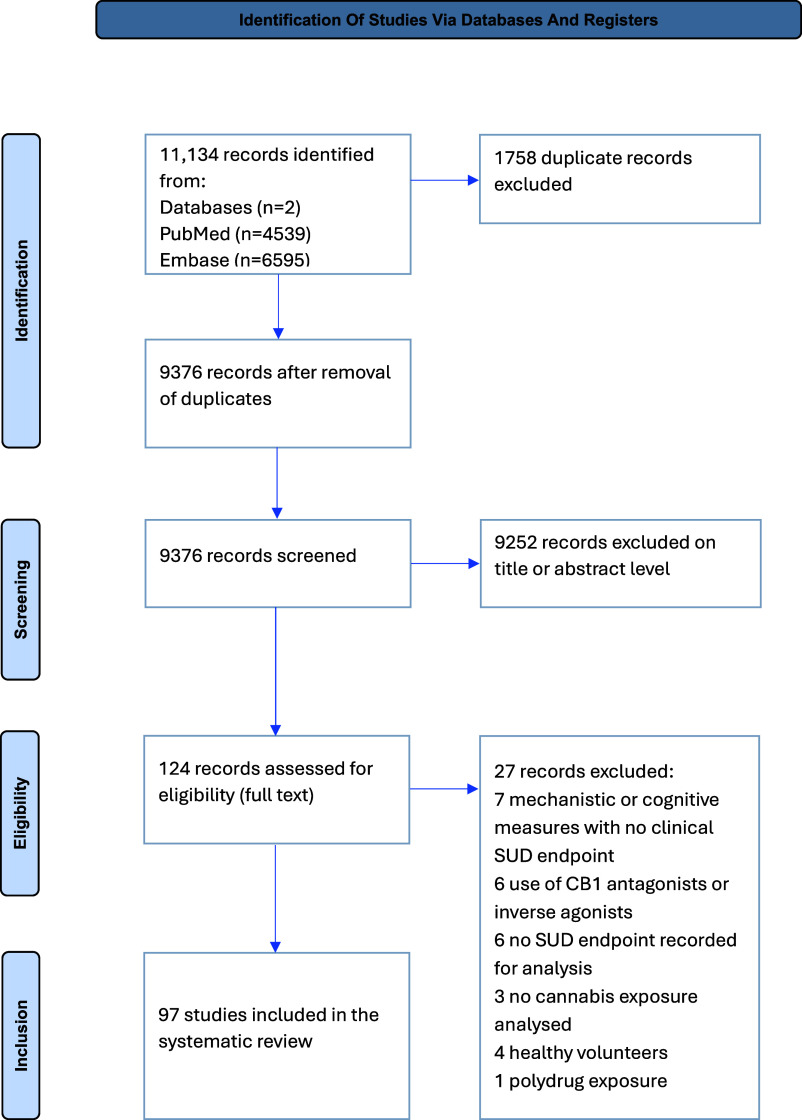
PRISMA 2020 flow diagram for the systematic review. PubMed: 4,539 records; Embase: 6,595 records; total: 11,134; duplicates removed: 1,758; records screened: 9,376; records excluded: 9,252; full-text articles assessed: 124; articles excluded with reasons: 27; studies included: 97. Figure 1. long description.

We conducted a systematic review aligned with the Preferred Reporting Items for Systematic Reviews and Meta-Analyses (PRISMA) statement [19], with a structured narrative synthesis following Synthesis Without Meta-analysis (SWiM) guidance [20]. The protocol was prospectively registered with PROSPERO (CRD420251151193). Searches covered PubMed and Embase from 1 January 1975 to 30 June 2025. These two databases were selected because they jointly provide near-complete coverage of the biomedical and clinical addiction literature, complemented by the additional backward and forward citation searching undertaken for all included records. Full strategies, field tags, and de-duplication procedures are reported in the Supplement. Two reviewers (DZD, AAZD) independently screened titles and abstracts, followed by full-text screening in duplicate. Inter-rater reliability for eligibility decisions was high (Cohen’s *κ* = 0.88, 96.0% agreement), and remaining disagreements were resolved by consensus. Trial registries were checked for ongoing or unpublished trials. No language restrictions were applied. Translations were arranged when required. No artificial intelligence tools were used at any stage of searching, screening or data extraction. In total, 124 full-text articles were assessed for eligibility, and 97 studies were included (Figure 1).

Eligible studies were original human research evaluating cannabis or cannabinoids as a specific exposure in relation to clinically relevant outcomes for SUDs. We considered randomized and non-randomized interventional designs and observational designs, provided that cannabis exposure was analyzed as a distinct variable rather than embedded in an undifferentiated illicit drugs composite. Observational eligibility was not restricted by subtype, and case–control designs were therefore admissible. One identified study [21] carried a case–control descriptor and was the only study so described. Because its groups were defined by cannabis exposure status rather than by outcome, and all measures were obtained concurrently at a single time point, it constitutes an analytical cross-sectional comparison rather than a case–control study in the epidemiological sense. It was accordingly classified within the cross-sectional stratum and appraised with the Joanna Briggs Institute (JBI) analytical cross-sectional tool. Studies without a cannabis-specific analysis were excluded. Where reports addressed several substances, a study was included for a given SUD only if that SUD had an analyzable cannabis-SUD endpoint association. We excluded animal and in-vitro studies, studies centered on cannabinoid receptor type 1 (CB1) antagonists or inverse agonists, healthy-volunteer experiments lacking SUD relevance, studies reporting only cognitive or laboratory measures without SUD endpoints, studies in which cannabis exposure could not be separated from polydrug exposure, narrative reviews, editorials, and protocols. When multiple publications described the same cohort, we linked them at the cohort level to avoid double counting.

### Outcomes and data extraction

Primary outcomes, prespecified and coded uniformly across designs following the SUD framework, were treatment retention, relapse, abstinence, craving, withdrawal severity, and consumption. Each outcome was mapped to its target SUD. Laboratory or mechanistic readouts were recorded for context but did not determine study-level efficacy unless designated as primary clinical endpoints by the original authors. For interventional trials, we extracted allocation, masking, intervention, comparator, timing, and outcome definitions. For observational studies, we captured the exposure definition and verified that cannabis was modeled as a separate exposure. Two reviewers (DZD, AAZD) independently extracted data with a piloted form and cross-checked entries. Inter-rater reliability for the coding of eligible studies was high (Cohen’s κ 0.84, 90.2% agreement), and discrepancies were resolved by discussion. Extracted items included study identifiers, setting and population, design, sample size, SUD category, exposure and outcome definitions, verification methods, follow-up duration, and statistical approach.

### Data analysis

Given substantial heterogeneity in design, exposure definitions, comparators, and timing, meta-analysis was not undertaken. Instead, synthesis proceeded narratively using prespecified disorder and endpoint strata. To retain quantitative detail without pooling incommensurable estimates, the effect sizes and confidence intervals reported by individual studies, where available, are compiled at the study level in a supplementary table organized by disorder and endpoint. Direction-of-effect classifications, reported as Beneficial, No Significant Effect, Mixed/Partial, or Harmful/Inferior, were applied consistently across studies, and findings were integrated within an endpoint-centered framework that incorporates design-based weighting for figures and summaries. Where studies reported on participants who used more than one substance, we extracted and synthesized the analysis isolating cannabis exposure from the other substances, in order to minimize confounding from polysubstance use.

To enhance interpretability across heterogeneous designs, we applied a tier-weighting framework, where each study received a base weight by tier type: RCT (1.00), prospective cohort (0.70), retrospective cohort (0.60), cross-sectional (0.45), case series (0.35), and qualitative (0.25). These values reflect the conventional evidence hierarchy [22], with decrements calibrated to the progressive loss of internal validity from randomization through self-selected exposure to cross-sectional measurement. The specific weights were compiled by the authors for this review and have not been formally validated as an instrument. They function as ordinal scaling aids for synthesis and figure construction, not as probability-based analytic weights, and do not replace study-level results. For each disorder and endpoint, the tier-weighted total within a direction-of-effect category was obtained by assigning each contributing endpoint instance the base weight of its study design and summing these weights across the instances in that category. Raw instance counts were retained alongside these totals and, to enable comparison across disorders, the direction-of-effect distribution within each disorder was additionally expressed as a percentage of that disorder’s total endpoint instances. We mapped designs to risk of bias (RoB) tools, applying RoB 2 to RCTs [23], Risk Of Bias In Non-randomized Studies of Interventions (ROBINS-I) to non-randomized comparative and cohort studies [24], JBI Cross-Sectional to analytical cross-sectional studies [25], JBI Case Series to case series [26], and JBI Qualitative to qualitative designs [27]. Confidence in the evidence for each disorder and endpoint was summarized using a simplified assessment appropriate to narrative synthesis without pooling, combining the strength of the contributing study designs, the risk of bias, and the consistency of the direction of effect, and it is reported with the risk of bias assessments in the Supplement [20, 22]. Where overlapping cohorts produced multiple reports, we kept analytically distinct, non-overlapping outcomes while assigning cohort-level participant counts to the most comprehensive report.

### Role of the funding source

None to declare.

## Results

After full-text assessment of 124 records, 97 studies met inclusion criteria (Figure 1), describing 41,954 unique participants (35.1% female). Publications spanned 1991–2025 and comprised 17 RCTs, 31 prospective cohort studies, 16 retrospective cohort studies, 20 cross-sectional studies, 4 case reports, and 9 qualitative studies (Table 1). These 97 studies contributed 195 endpoint instances across the six prespecified SUD endpoints. Of these, 89 (45.6%) were classified as Beneficial, 80 (41.0%) as No Significant Effect, 12 (6.2%) as Mixed/Partial, and 14 (7.2%) as Harmful/Inferior.

**Table 1.** Characteristics of included studies Table 1. long description.

Study	Study design	Sample size (female %)	Mean age (SD), years	Form, dose, route	Direction-of-effect: SUD endpoint (SUD)	Outcome
Gossop et al. [28]	Cross-sectional retrospective self-report survey	50 (30%)	29.4 (NA) range 21–40	NA	Mixed/Partial: Withdrawal severity (OUD)	22 participants reported using cannabis. 12 said cannabis made withdrawal worse. 6 said it reduced distress.
Saxon et al. [29]	Retrospective analysis of clinical data	98 (0%)	43.9 (8.3) range 32–77	NA	No significant effect: Abstinence (OUD) No significant effect: Retention (OUD)	There was no significant difference in the use of other drugs between THC +ve (median 6.5%) and THC −ve (median 6.3%) patients (*z* = −0.48, NS). Cannabis status did not impact treatment retention.
Nirenberg et al. [30]	Retrospective chart review	70 (1.4%)	39.0 (NA)	NA	No significant effect: Consumption (OUD)	Cannabis use was not associated with higher rates of opiate-positive urine screens (mean: 18% vs. 11%; F [1,68]=0.90, *p* = 0.35).
Saxon et al. [31]	Longitudinal analysis within RCT	353 (38.2%)	38.2 (8.0)	NA	No significant effect: Retention (OUD) No significant effect: Consumption (OUD)	Pre-treatment cannabis frequency did not predict illicit opioid use during treatment (Beta = 0.05, NS) and was not a significant predictor of retention (Cox regression: Exp(B) = 1.08, 95% CI 0.97–1.20, NS).
Budney et al. [32]	Prospective cohort (secondary analysis of RCT)	107 (37%)	34.0 (7.9)	NA, inhaled	No significant effect: Retention (OUD) No significant effect: Abstinence (OUD)	There were no significant differences between marijuana users and non-users regarding treatment retention (65% vs. 60% weeks) or opioid abstinence (8.4 vs. 8.5 weeks).
Wasserman et al. [33]	Prospective observational study	74 (40.5%)	42.8 (9.3) range 19–67	NA	Harmful/Inferior: Relapse (OUD)	Baseline cannabis use significantly increased the risk of a heroin lapse during the 8-week study (X^2^(1, *N* = 74) = 8.39, P < 0.004) and at the 6-month follow-up (X^2^(1, *N* = 69) = 7.90, P < 0.005). It also significantly predicted lapse when analyzed as a time-dependent variable (P < 0.006).
Best et al. [34]	Cross-sectional survey	200 (30%)	32.0 (8.2) range 16–54	NA, inhaled	Beneficial: Consumption (OUD)	Daily cannabis users reported significantly fewer days of heroin use in the previous month (mean 0.8 days) compared to occasional users (1.6 days) and non-users (5.8 days) (*F* = 11.07, p < 0.0001). However, anxiety (9.3 vs. 6.7) and depression (9.7 vs. 7.6) were higher in daily users (p < 0.01).
Labigalini et al. [35]	Prospective case series	25 (0%)	NA range 16–28	3–4 cigarettes daily, inhaled	Beneficial: Abstinence (CoUD) Beneficial: Craving (CoUD) Beneficial: Withdrawal severity (CoUD)	68% ceased crack use (mean time to cessation: 5.2 weeks) and reported reduced craving and the “overpowering urge” to use crack. Participants reported declines in anxiety and withdrawal symptoms and improved sleep. Significant physical recovery was observed (average weight from 52.3 kg to 64.1 kg at 9 months).
Church et al. [36]	Prospective cohort (within open-label trial)	47 (23%)	33.6 (9.3) range 20–54	NA	Mixed/Partial: Abstinence (OUD) No significant effect: Retention (OUD)	Intermittent cannabis users had significantly lower opiate-positive urines (15%) compared to abstainers (60%) and heavy users (71.4%) (*F* = 9.381, p < 0.001). However, continuous linear correlations between cannabis +ve urines and opioid outcomes were non-significant. No significant difference in retention across cannabis-use groups (*F* = 1.932, *p* = 0.159).
Dreher [37]	Qualitative ethnographic longitudinal study	33 (100%)	NA	Cigarettes (spliffs), inhaled	Beneficial: Craving (CoUD) Beneficial: Relapse (CoUD) Beneficial: Abstinence (CoUD)	Users reported reduced the urge to use crack, describing cannabis as “the cheapest, most effective and readily available therapy.” 13 of the 14 (92.8%) women who stopped crack use attributed their success to ganja.
Epstein & Preston[38]	Retrospective pooled cohort (secondary analysis of RCT)	408 (40%)	38.5 (6.1) range 21–57	NA	No significant effect: Retention (OUD) No significant effect: Abstinence (OUD) No significant effect: Abstinence (CoUD) No significant effect: Relapse (OUD)	Cannabis use category not associated with treatment retention in any of the three trials (p values .62–.79) and did not predict % of opiate- or cocaine- + ve urines (r^2^ < .03, NS). Among heroin-abstinent patients, cannabis use did not significantly increase the hazard of heroin lapse (hazard ratios ~1.20–1.54; p ≥ .095).
Weizman et al. [39]	Prospective cohort study	196 (NA)	37.4 (7.1)^a^	NA	No significant effect: Retention (OUD) No significant effect: Consumption (OUD)	Cannabis users did not leave treatment earlier (*B* = −0.17, *p* = 0.21). At 1 year, they were not found to consume more heroin than non-users (ANOVA: NS).
Aharonovich et al. [40]	Prospective cohort study	250 (34%)	36.9 (9.2)	NA	Harmful/Inferior: Relapse (AUD) No significant effect: Relapse (OUD) No significant effect: Relapse (CoUD)	Cannabis use significantly increased relapse to alcohol (HR 5.09, 95% CI 1.44–15.72, p < 0.05). For cocaine, it was borderline but NS (HR 5.57,95% CI 0.97–8.94) and for opioids it was NS (HR 2.18, 95% CI 0.22–2.54, NS).
Hermann et al. [41]	Cross-sectional survey	89 (28%)	34 (6.0)	NA	Mixed/Partial: Withdrawal severity (OUD)	Patients rated cannabis efficacy in alleviating opioid withdrawal as 3.6 ± 1.0 on a 5-point scale (1 = very good, 5 = no reduction). Some reported benefit, 15% no effect, and 37.5% worsening of withdrawal.
Li et al. [21]	Cross-sectional case–control study	26 (38.5%)	36.8 (6.2)	NA	No significant effect: Craving (CoUD)	While stress imagery significantly increased cocaine craving compared to neutral imagery (*F* = 16.224, *p* = 0.000), the magnitude of this increase between cannabis users and non-users was NS (group × condition interaction *F* = 0.200, *p* = 0.656).
Peles et al. [42]	Prospective cohort study	492 (27.2%)	36.7 (8.5) range 18–67	NA	No significant effect: Retention (OUD)	Association between cannabis use at admission and 1-year retention rates was NS (81.8% for THC + ve vs. 74.1% for THC−ve; *p* = 0.3, Fisher’s Exact). In multivariate Cox regression for long-term cumulative retention (up to 11 years), cannabis use as a predictor was NS, unlike opiate abuse, methadone dose, and parenthood status.
Nava et al. [43]	Prospective cohort study	121 (14%)	28.9 (6.0)	NA, 8.30 joints/week, inhaled	No significant effect: Withdrawal severity (OUD) No significant effect: Craving (OUD) No significant effect: Consumption (OUD) No significant effect: Retention (OUD)	Opioid withdrawal scores decreased in both cannabis users (*z* = −7.58, p < 0.001) and non-users (*z* = −7.30, p < 0.001), marking NS. Craving declined in both groups (Users: *z* = −5.24; Non-users: *z* = −5.02; both p < 0.001), thus NS. Less opioid +ve urines in both groups (users: *z* = −3.42; non-users: *z* = −3.18; p < 0.001), so NS. NS difference in retention between groups (Kaplan–Meier).
Reiman [44]	Cross-sectional survey	130 (25%)	39.9 (12.9) range 18–78	NA	Beneficial: Withdrawal severity (AUD) Beneficial: Consumption (AUD)	50% substituting cannabis for alcohol. 43% reported “fewer withdrawal symptoms.” 63% cited “fewer side effects” and 55% cited “better symptom management.”
Schiff et al. [45]	Retrospective longitudinal data review	2683 (12.3%)	43.3 (8.53)	NA	Beneficial: Retention (OUD)	In a logistic regression model, cannabis use significantly increased the likelihood of 100% retention during the 13-month study period (OR 1.43, 95% CI 1.15–1.78, p < .001).
Oliveira & Nappo [46]	Qualitative ethnographic study	62 (25.8%)	NA	NA, inhaled	Beneficial: Craving (CoUD) Beneficial: Withdrawal severity (CoUD)	Interviewees used cannabis as a “palliative” to “calm down” after crack use, diminishing craving. They said it also reduced “other symptoms associated with the syndrome of abstinence from crack” like agitation and paranoia.
Raby et al. [47]	Prospective cohort (secondary analysis of RCT)	63 (17%)	35.5 (9.2)	NA	Beneficial: Retention (OUD)	Intermittent cannabis users had superior retention (median 133 days) compared to abstinent (35 days) and consistent users (35 days) (*p* = 0.002). In Cox regression, intermittent use significantly reduced dropout (HR 0.23, 95% CI 0.09–0.57, $p = 0.001$). Consistent/heavy use did not improve retention overall.
Reiman [48]	Cross-sectional survey	350 (31.6%)	39.43 (NA)	NA	Beneficial: Withdrawal severity (AUD) Beneficial: Consumption (AUD)	34% used cannabis as a substitute due to “less withdrawal potential.” 65% cited “less adverse side effects” and 57.4% “better symptom management.” 40% reported substituting for alcohol, reducing consumption.
Ribeiro et al. [49]	Qualitative interview study	28 (28.6%)	32 (NA) range 20–47	NA, inhaled	Mixed/Partial: Craving (CoUD) Beneficial: Withdrawal severity (CoUD)	Cannabis used to reduce cravings, mixing it with crack or smoking it after to avoid compulsive crack use. However, some describe the mix as part of a binge ritual that prolongs the session. It helped with control of withdrawal symptoms and paranoia.
Alessi et al. [50]	Retrospective cohort (secondary analysis of RCT)	393 (29.3%)	36.1 (8.1)	NA	Mixed/Partial: Retention (CoUD) No significant effect: Abstinence (OUD) No significant effect: Abstinence (AUD) No significant effect: Abstinence (CoUD)	Shorter retention for cannabis users (mean: 3.7 weeks vs. 5.6 weeks). However, contingency management improved it to 7.3 weeks (Interaction effect F(1,383) = 4.2, *p* = 0.04). NS of cannabis on longest abstinence duration from cocaine, opioids, alcohol (*p* = 0.95), or the % of −ve samples (*p* = 0.81).
Andrade et al. [51]	Qualitative interview study	22 (31.8%)	29 (NA) range 18–52	Pitilho, inhaled	Beneficial: Craving (CoUD)	Crack + cannabis (pitilho), used by four interviewees, decreased the craving for more crack and reduced associated paranoia, allowing for a more controlled pattern of use.
Chaves et al. [52]	Qualitative semi-structured interview study	40 (50%)	NA range 18–50	NA	Beneficial: Craving (CoUD)	Respondents reported using “depressant agents” such as alcohol and cannabis to “induce sleep” and alleviate craving.
Roux et al. [53]	Prospective longitudinal cohort study	235 (30.6%)	34 [31–37]^b^	NA	Harmful/Inferior: Abstinence (OUD) Harmful/Inferior: Relapse (OUD)	Daily cannabis use was independently associated with more non-medical use of opioids (adjusted coefficient = 0.28, 95% CI 0.08; 0.47, *p* = 0.01).
Chaudhry et al. [54]	Retrospective cohort study	142 (6.3%)	26.4 (NA) range 17–61	NA	No significant effect: Retention (OUD)	In univariate analysis, cannabis use was associated with lower retention (19–26% progressed to stage III vs. 43% of non-users, $p = 0.04$). However, in the multivariate logistic regression model adjusting for confounders, it was a NS predictor of retention.
Green et al. [55]	Retrospective cohort (secondary analysis of RCT)	177 (NA)	NA	NA	Mixed/Partial: Abstinence (CoUD)	For cocaine abstinence, in the levodopa group, each day of cannabis use predicted a 5.4% decrease, while in the placebo group it predicted a 4.9% increase.
Somers & O’Connor [56]	Retrospective cohort study	117 (35%)	33.3 (6.9)	NA	No significant effect: Abstinence (OUD)	Urine cannabis showed NS association with “good urinary heroin outcome” (heroin −ve or < 20% + ve urines). The unadjusted OR 0.84 (95% CI 0.57–1.25); adjusted OR 0.32 (95% CI 0.07–1.57).
Hill et al. [57]	Prospective cohort (secondary analysis of RCT)	152 (NA)	Range 15–21	NA	No significant effect: Abstinence (OUD) No significant effect: Retention (OUD)	Cannabis use (whether measured by age of initiation, past 30-day frequency, or concurrent use) had NS association with opioid use during the 12-week trial (p > 0.05). NS association with treatment completion: abstainers (48%), occasional (61%), daily users (56%) (*p* = 0.38).
Lucas et al. [58]	Cross-sectional survey	404 (32.9%)	44.12 (NA) range 17–71	NA	Beneficial: Craving (OUD) Beneficial: Craving (AUD) Beneficial: Craving (CoUD) Beneficial: Withdrawal severity (OUD) Beneficial: Withdrawal severity (AUD) Beneficial: Withdrawal severity (CoUD) Beneficial: Relapse (OUD) Beneficial: Relapse (AUD) Beneficial: Relapse (CoUD)	75.5% reported substituting cannabis for at least one other substance, to cope with urges. 67.7% cited less withdrawal. Cannabis considered a potential “exit drug,” reducing continued SUD.
Morgan et al. [59]	RCT, double-blind, placebo-controlled pilot	24 (50%)	28.0 (5.3) range 18–35	CBD, inhaler prn, inhaled	Beneficial: Consumption (TUD) No significant effect: Craving (TUD)	Over 7 days, the CBD group significantly reduced cigarettes smoked by ~40% (*p* = 0.002), while the placebo group showed no change. Maintained at 2 weeks (*p* = 0.034). The time × treatment interaction was borderline significant (F [2,42]=3.12, *p* = 0.054). NS difference between CBD and placebo in craving scores (TCQ and daily VAS).
Potter et al. [60]	Prospective cohort (secondary analysis of RCT)	1269 (32.2%)	37.4 (11.1)	NA	Beneficial: Abstinence (OUD) No significant effect: Retention (OUD)	Cannabis use was significantly associated with higher odds of opioid abstinence at the 24-week treatment phase end (aOR 0.48; 95% CI 0.25–0.92). However, NS as predictor for treatment retention or time-to-dropout.
Scavone et al. [61]	Retrospective chart analysis	91 (60.4%)	39.37 (11.29) range 20–62	NA	Beneficial: Withdrawal severity (OUD) No significant effect: Abstinence (OUD) No significant effect: Retention (OUD)	NS association between cannabis use and rates of opiate +ve urine screens during induction (*r* = 0.104, *p* = 0.332) or stabilization phases. No impact methadone blocking dose, time to stabilization, or treatment attendance. However, in a subset analysis, cannabis users exhibited lower opiate withdrawal scores (*p* = 0.006).
Hser et al. [62]	Prospective cohort (secondary analysis of RCT)	1267 (32.0%)	37.4 (11.1)	NA	Harmful/Inferior: Retention (OUD)	Cannabinoid positivity during the 24-week trial was significantly associated with a higher risk of dropout (HR = 2.10, 95% CI 1.62–2.71, p < 0.01). Authors noted contrast with previous studies and hypothesized that higher potency cannabis might be “more destabilizing.”
Lions et al. [63]	Prospective cohort (secondary analysis of RCT)	158 (15.2%)	33 [28–39]^b^	NA	No significant effect: Relapse (OUD)	While daily cannabis use was associated with a higher risk of relapse in univariate analysis (OR 2.81, 95% CI 1.22–6.48), this association was NS in the final multivariate model. Alcohol or cocaine use, living with a heroin user, and the patient–physician relationship were independent predictors of the outcome.
Matson et al. [64]	Retrospective cohort (chart review)	103 (49.5%)	19.2 (1.6) range 15.7–24.4	NA	Harmful/Inferior: Retention (OUD)	In Cox regression analysis, +ve urine for THC predicted a higher hazard of not returning to treatment (HR 1.73, 95% CI 1.14–2.63, p < 0.001).
Viola et al. [65]	Prospective cohort study	93 (100%)	28.4 (6.5)	NA	Harmful/Inferior: Withdrawal severity (CoUD) Harmful/Inferior: Craving (CoUD) Harmful/Inferior: Relapse (CoUD)	Early-onset cannabis users had higher cocaine craving scores at day 14 of detox (5.25 vs. 2.48, *p* = 0.004) and were more likely to experience an increase in withdrawal severity (46.7% vs. 16%). Long-term use was an independent predictor of higher rehospitalization rates for cocaine dependence at 2.5 years (Beta = 0.28, *p* = 0.011).
Abrahamsson et al. [66]	Prospective cohort study	44 (11.4%)	35 (NA) range 20–55	NA	No significant effect: Retention (OUD) No significant effect: Abstinence (OUD)	Past 30-day cannabis use predicted successful transfer in univariate analysis (5.2 vs. 10.4 days, *p* = 0.06) but NS in the multivariate model (*p* = 0.27). Similarly, cannabis use was NS between opiate –ve vs. opiate +ve urine samples (5.9 vs. 8.6 days).
Bisaga et al. [67]	RCT, double-blind, placebo-controlled	60 (15%)	38 (11.3)	Dronabinol, 30 mg/day, oral	Beneficial: Withdrawal severity (OUD) No significant effect: Retention (OUD) No significant effect: Abstinence (OUD) No significant effect: Craving (OUD)	Dronabinol (30 mg/day) significantly reduced the severity of subjective opioid withdrawal (SOWS) during the inpatient detoxification phase vs. placebo (*p* = 0.006), particularly before naltrexone induction. However, NS improvement in rates of successful induction onto extended release naltrexone (66% vs. 55%) or retention at 8 weeks (35% vs. 35%).
Epstein & Preston [68]	Prospective cohort (secondary analysis of RCT)	116 (47%)	39 (7.3) range 21–59	NA, inhaled	No significant effect: Withdrawal severity (OUD)	NS difference in overall opioid withdrawal scores between cannabis users (*n* = 46) and non-users (*n* = 70) (F [1,104]=0.33, *p* = 0.57). No evidence of withdrawal reduction in time-lagged analyses in users in the subsequent week (effect size *r* = 0.01, *p* = 0.69).
Gonçalves & Nappo [69]	Qualitative interview study	27 (NA)	25.9 (NA) range 19–49	NA, inhaled	Beneficial: Craving (CoUD) Beneficial: Relapse (CoUD)	Cannabis + crack (“mesclado”) reduced paranoia, aggression, craving, and extended the interval between crack doses (from 5 to 10 minutes to 1–2 hours), thereby reducing compulsive seeking behavior and improving quality of life (sleep, appetite).
Lau et al. [70]	Qualitative interview study	97 (36%)	58 range 48–68	NA, inhaled, oral	Beneficial: Craving (OUD) Beneficial: Craving (AUD) Beneficial: Craving (CoUD) Beneficial: Relapse (OUD) Beneficial: Relapse (AUD) Beneficial: Relapse (CoUD) Beneficial: Abstinence (OUD) Beneficial: Abstinence (AUD) Beneficial: Abstinence (CoUD)	Cannabis is used as a substitute for alcohol, cocaine, and opioids. Described as effective for reducing cravings, managing withdrawal symptoms, and maintaining abstinence.
Levine et al. [71]	Retrospective chart review	290 (40.3%)	50.3 (8.9)	NA	Harmful/Inferior: Retention (OUD) No significant effect: Abstinence (OUD)	+ve cannabinoid urine in month 1 was a significant predictor of treatment dropout (retention <1 year) for both genders (males: OR 5.00, 95% CI 1.61–14.29; females: OR 9.09, 95% CI 2.33–33.33). However, early cannabis use did not predict long-term opioid abstinence for either gender in linear regression models.
Mayet et al. [72]	Prospective cohort (secondary analysis of RCT)	176 (15.3%)	33 [27–38]^b^	NA	No significant effect: Withdrawal severity (OUD)	NS association between the number of opioid withdrawal symptoms (OOWS) and either non-daily (*p* = 0.80) or daily (*p* = 0.67) cannabis use in univariate mixed models.
Teixeira et al. [73]	Qualitative interview study	5 (20%)	31.8 (NA) range 22–42	NA, inhaled	Beneficial: Craving (CoUD) Beneficial: Withdrawal severity (CoUD)	Participants used cannabis after crack to manage the “fissure” (intense craving) and harsh comedown symptoms (insomnia, lack of appetite) of crack binges.
Dayal et al. [74]	Retrospective cohort	140 (0.7%)	31.65 (8.57)	NA	No significant effect: Retention (OUD)	Cannabis use at intake was associated with shorter retention in bivariate analysis (*z* = 2.06, *p* = 0.03). However, in multivariate logistic regression models predicting retention at 90 days (OR 0.46, *p* = 0.49) and 180 days (OR 0.10, *p* = 0.17), it was NS as a predictor when controlling for employment, marital status, and opioid history.
Håkansson et al. [75]	Prospective cohort study	36 (11.1%)	33 [29–42]^b^	NA	No significant effect: Retention (OUD)	NS association between cannabis use and 9-month retention. Neither cannabis +ve urines during interim phase (*p* = 0.297) nor during the full-scale treatment phase (*p* = 0.965) differed significantly between those retained and those who dropped out.
Lofwall et al. [76]	RCT, double-blind, placebo-controlled, within-subject	12 (50%)	31.3 (5.2)	Dronabinol, 5–30 mg, oral	Beneficial: Withdrawal severity (OUD) Beneficial: Craving (OUD)	Dronabinol 20 and 30 mg significantly suppressed withdrawal symptoms vs. placebo (observer-rated antagonist scores 4.0–4.2 vs. 6.7). Dronabinol 20 mg significantly reduced desire for opiates compared to placebo. However, higher doses produced tachycardia and anxiety, limiting clinical utility.
Proctor et al. [77]	Retrospective multi-site chart review	2410 (40.4%)	34.5 (10.77) range 18–82	NA	Harmful/Inferior: Relapse (OUD)	Cannabis use was significantly associated with ongoing opioid use. At 3 months, doubled the odds of being opioid +ve at 6 months (aOR 2.03, 95% CI 1.03–3.98). At 9 months, it was a strong independent predictor of opioid relapse at 12 months (aOR 5.19, 95% CI 1.26–21.47).
Dayal & Balhara [78]	Retrospective cohort study	68 (NA)	22.35 (2.47)	NA	No significant effect: Retention (OUD)	Past-month cannabis use (reported by 16.2% of participants) as a predictor of treatment retention was NS in multivariate analysis, whereas family substance use, buprenorphine dose, and injection drug use were significant predictors.
Franklyn et al. [79]	Retrospective cohort study	644 (40.4%)	31.0 (10.7)	NA	Harmful/Inferior: Retention (OUD)	Baseline cannabis use (50.9% of sample) was associated with significantly lower 1-year retention (61.0% vs. 70.9%) and increased risk of dropout (aHR 1.39; 95% CI 1.06–1.83). Heavy cannabis use (≥75% + ve urine screens) further increased dropout risk (aHR 1.48; 95% CI 1.13–1.93), with sex-stratified analysis revealing specific risks for female baseline users and male heavy users.
Giasson-Gariépy et al. [80]	Cross-sectional comparative study	28 (0%)	44.17 (9.43)	NA	Harmful/Inferior: Craving (CoUD)	Cue-induced cocaine craving was successfully elicited in cannabis users and non-users with NS difference in magnitude. However, users exhibited a significant progressive increase in pre-cue baseline craving scores across repeated blocks (time × group interaction: *F* = 3.3, *p* = 0.023), suggesting a deficit in craving decay.
Sociás et al. [81]	Prospective cohort (longitudinal analysis)	122 (27%)	29.1 (24–30.1)^b^	NA	Beneficial: Consumption (CoUD)	Cannabis use to control crack was significantly associated with reduced crack use compared to baseline (AOR 1.89; 95% CI 1.01–3.45).
Zielinski et al. [82]	Cross-sectional study	777 (46.7%)	38.05 (11.11)	NA, 1.31 g/day	Mixed/Partial: Relapse (OUD)	Cannabis association with opioid use was NS (OR 1.16; 95% CI 0.77–1.75). However, in sex-stratified analyses, it was significantly associated with increased opioid use in women (OR 1.82; 95% CI 1.18–2.82) but not in men (OR 1.11; 95% CI 0.73–1.69), although the sex-by-cannabis interaction was not statistically significant (*p* = 0.17). Heaviness of cannabis use was not associated with opioid use in either sex.
Bagra et al. [83]	Cross-sectional comparative study	100 (0%)	43.9 (11.07)	2 cigarettes/day, inhaled	No significant effect: Abstinence (OUD) No significant effect: Craving (OUD) No significant effect: Withdrawal severity (OUD)	Cannabis use (past 3 months) was NS associated with opioid use (17.1% vs. 13.8%, *p* = 0.66), craving (22.9% vs. 16.9%, *p* = 0.65), or withdrawal symptoms (22.9% vs. 13.8%, *p* = 0.75) compared to non-use, despite cannabis users receiving a significantly lower mean daily buprenorphine dose (7.9 vs. 8.9 mg, *p* = 0.04).
Hindocha et al. [84]	RCT, double-blind, placebo-controlled crossover	30 (46.7%)	28.07 (8.66)	CBD, 800 mg, oral	No significant effect: Craving (TUD) No significant effect: Withdrawal severity (TUD)	CBD 800 mg presented NS reduction in tobacco craving (Bayes factor = 7.08) or withdrawal symptoms (Bayes factor = 6.95) vs. placebo during acute abstinence, despite significantly reversing automatic attentional bias toward cigarette cues (*p* = 0.007, *d* = 0.704) and reducing explicit pleasantness of cigarette stimuli (*p* = 0.011, *d* = 0.514).
Klimas et al. [85]	Prospective cohort (longitudinal analysis)	823 (39.9%)	42 (35.8–47.9)^b^	NA	No significant effect: Retention (OUD)	Daily cannabis use was not associated with OUD treatment discontinuation (unadjusted HR 0.84; 95% CI 0.64–1.11; *p* = 0.23) and was a NS predictor in multivariable analyses, whereas daily heroin injection and homelessness significantly increased discontinuation risk.
Peles et al. [86]	Retrospective cohort study	890 (25.2%)	NA	NA	No significant effect: Retention (OUD)	Urines +ve for cannabis on admission showed NS association with treatment retention (*p* = 0.8), whereas methadone dose ≥100 mg/day and absence of benzodiazepine or opioid use after 1 year significantly predicted longer retention.
Eastwood et al. [87]	Prospective cohort study	7717 (NA)	NA	NA	Mixed/Partial: Retention (OUD)	Cannabis use showed mixed associations with treatment completion. A “low and decreasing” cannabis trajectory predicted higher success among patients with “rapid decreasing” heroin use (AOR 2.39; 95% CI 1.29–4.40) but lower odds in those with “decreasing then increasing” heroin use (AOR 0.50; 95% CI 0.28–0.92). A “high and increasing” cannabis trajectory had NS association with retention.
Hurd et al. [88]	RCT, double-blind, placebo-controlled parallel-group	42 (16.7%)	49.8 (9.2)	Epidiolex, 400–800 mg, oral	Beneficial: Craving (OUD)	42 abstinent OUD participants had lower cue-induced craving (VAS-C) vs. placebo (*F* = 5.74, *p* = 0.0047), with effects persisting 7 days after final CBD dose. It also blunted cue-induced anxiety (VAS-A) and physiological stress reactivity (heart rate, cortisol).
Lucas et al. [89]	Cross-sectional survey	2032 (37.3%)	40 (NA)	Various, 1.5 g/day, various	Beneficial: Abstinence (OUD) Beneficial: Abstinence (AUD) Beneficial: Abstinence (TUD) Beneficial: Consumption (OUD) Beneficial: Consumption (AUD) Beneficial: Consumption (TUD)	69.1% substituted cannabis for SUDs. Among those substituting for opioids, 59.3% reported complete abstinence and 18.4% reported a ≥75% reduction. For alcohol, 30.9% reported abstinence and 36.7% a ≥75% reduction. For tobacco, 50.7% reported abstinence.
Oliveira et al. [90]	Prospective cohort study	123 (14.6%)	30.2 (7.2)	NA, inhaled	No significant effect: Relapse (CoUD) No significant effect: Retention (CoUD)	6 months post-discharge, CoUD participants with concomitant heavy cannabis use demonstrated associated as NS with relapse to cocaine or treatment retention at 1, 3, or 6 months compared to cocaine-only users (p > 0.1).
Shams et al. [91]	Cross-sectional study	672 (45.8%)	38.77 (11.1)	NA, 1.15 g/day	No significant effect: Abstinence (OUD)	Cannabis use was associated with significantly lower self-reported heroin use in the past 30 days (aOR 0.45; 95% CI 0.24–0.86; *p* = 0.016), but NS association with improved abstinence on urine toxicology (aOR 1.37; 95% CI 0.97–1.93; *p* = 0.077).
Bergeria et al. [92]	Cross-sectional survey	200 (44%)	33.2 (9.6)	NA	Beneficial: Withdrawal severity (OUD)	Self-reported withdrawal severity was significantly lower on days with cannabis use compared to days without (SOWS: 16.2 vs. 27.8, p < .05; VAS: 35.3 vs. 64.5, p < .05). Anxiety (76.2%), tremors (54.1%), and trouble sleeping (48.4%) were most improved.
Karoly et al. [93]	Cross-sectional online survey	533 (43.7%)	34.9 (14.3)	Various, NA, various	Mixed/Partial: Consumption (AUD)	Among cannabis users who consume alcohol, those using cannabis to treat a medical condition reported fewer drinking days compared to non-medical users (2.04 vs. 2.57; *p* = 0.024) but NS difference in drinks per occasion. Edible users using low-THC/high-CBD products reported fewer drinks per occasion (2.63 vs. 3.15 vs. 3.43; *p* = 0.019) and less frequent co-use (*p* = 0.027). Conversely, flower users using high-THC/low-CBD products consumed more alcohol on days of co-use (*p* = 0.020).
Valleriani et al. [94]	Qualitative ethnographic study	23 (57%)	46.7 (NA) range 21–65	Various, NA, various	Beneficial: Craving (OUD) Beneficial: Craving (CoUD) Beneficial: Craving (MUD) Beneficial: Withdrawal severity (OUD) Beneficial: Withdrawal severity (CoUD) Beneficial: Withdrawal severity (MUD) Beneficial: Consumption (OUD) Beneficial: Consumption (CoUD) Beneficial: Consumption (MUD)	74% used cannabis to reduce use of other drugs, to manage pain and withdrawal symptoms (“dope sickness”) from opioids and stimulants, and to reduce consumption of crack, methamphetamine, and fentanyl.
Viola et al. [95]	Prospective cohort	214 (100%)	33.42 (8.16)	NA	Harmful/Inferior: Withdrawal severity (CoUD)	Frequent recent cannabis use (>5 days in the past month) was associated with significantly higher cocaine withdrawal severity at discharge compared to non-use (*p* = 0.028, *d* = 0.43). Occasional cannabis use (1–5 days) was not associated with worse withdrawal.
Karoly et al. [96]	RCT, open-label, parallel-group	120 (39.2%)	33.2 (14.2)	Flower, 1–3 g/5 days, inhaled	Beneficial: Consumption (AUD)	The CBD-dominant group reported significantly fewer drinks per drinking day (β = 0.180; *p* = 0.031), fewer alcohol use days (β = 0.141; *p* = 0.035), and fewer alcohol and cannabis co-use days (β = 0.145; *p* = 0.035) compared to the THC and CBD + THC groups combined over the 5-day ad libitum period, covarying for baseline drinking.
Liu et al. [97]	Cross-sectional survey	2968 (39.6%)	46.7 (11.3)	NA	Beneficial: Consumption (CoUD)	Cocaine + cannabis users had a lower lifetime CoUD prevalence (22.2%) vs. cocaine-only users (26.9%). Also less likely to report “very high” cocaine quantity (19.1% vs. 24.7%), frequency (16.9% vs. 29.6%), and duration (14.2% vs. 21.0%). Mediation analysis revealed a significant indirect effect via quantity of cocaine use (indirect effect = −0.09, 95% CI [−0.19, −0.001]), indicating less risk of CoUD is mediated by lower use intensity.
Meneses-Gaya et al. [98]	RCT, double-blind, placebo-controlled	31 (0%)	32.9 (6.8)	CBD, 150 mg BD, oral	No significant effect: Craving (CoUD) No significant effect: Withdrawal severity (CoUD)	NS reduction in craving with CBD vs. placebo over a 10-day period (time × treatment interaction: F{10,230} = 0.489; *p* = 0.897). No superiority in reducing anxiety, depression, or sleep disturbances.
Meyer et al. [99]	Case report	1 (0%)	30 years	CBD, 400 mg, inhaled	Beneficial: Abstinence (CoUD)	CBD cigarettes and off-label methylphenidate led to complete abstinence from cocaine, verified by ˗ve urine screens at discharge, 6 weeks, and 12 weeks follow-up, with no readmissions.
Mok et al. [100]	Cross-sectional analysis of prospective cohorts	1936 (38%)	42 [29–53]^b^	NA	Beneficial: Withdrawal severity (OUD) Beneficial: Withdrawal severity (AUD) Beneficial: Withdrawal severity (CoUD) Beneficial: Consumption (OUD) Beneficial: Consumption (AUD) Beneficial: Consumption (CoUD)	22% (*n* = 425) reported using cannabis for harm reduction at least once; substituting for stimulants (50%), opioids (31%), and treating withdrawal (15%), indicating high cannabis use as a self-directed management strategy.
Mongeau-Pérusse et al. [101]	RCT, double-blind, placebo-controlled	78 (17.9%)	45.9 (11.2)	CBD, 800 mg/day, oral	No significant effect: Craving (CoUD) No significant effect: Withdrawal severity (CoUD) No significant effect: Relapse (CoUD) No significant effect: Consumption (CoUD)	NS reduction to drug-cue-induced craving (*P* = 0.069) or cocaine withdrawal symptoms during detox, nor did it delay time to relapse (*P* = 0.51) or reduce cocaine use days (*P* = 0.682) during the 12-week outpatient follow-up compared to placebo.
Rosic et al. [102]	Prospective cohort study	2315 (44.3%)	39.3 (10.9)	NA	Mixed/Partial: Abstinence (OUD) No significant effect: Craving (OUD) No significant effect: Withdrawal severity (OUD)	Past-month cannabis use (*n* = 1,178) vs. non-use (*n* = 1,135) was NS associated with abstinence at 3 months (OR 1.03; 95% CI 0.87–1.23; *p* = 0.703). However, daily use was significantly associated with lower opioid use (OR 0.60; 95% CI 0.46–0.78; p < 0.001) vs. occasional use, as was older age of onset (OR 0.97; *p* = 0.032) and reporting cannabis-related side effects (OR 0.66; *p* = 0.001). Few stated it helped with craving (6.9%) or withdrawal (8.3%), with 74.9% reporting no impact.
Shaw & Marcu [103]	Case report	1 (0%)	27 years	CBD, 600 mg/day, sublingual	Beneficial: Withdrawal severity (OUD) Beneficial: Craving (OUD) Beneficial: Abstinence (OUD)	Successfully managed acute withdrawal (COWS score reduced from 14 at Day 0 to 3 at Day 7) and facilitated transition to naltrexone induction on Day 10 without precipitated withdrawal or relapse.
Bunting et al. [104]	Prospective cohort (secondary analysis of RCT)	474 (30%)	33.66 (9.59)	NA	No significant effect: Relapse (OUD) No significant effect: Craving (OUD)	Neither pretreatment cannabis use nor cannabis use during the first 4 weeks of treatment was significantly associated with opioid relapse by week 24 or craving scores (all p > 0.05); however, cannabis use prevalence significantly increased from 11% to 17% during the first 4 weeks (*p* = 0.02).
Naji et al. [105]	Prospective cohort study	466 (46.1%)	38.59 (10.73)	NA	No significant effect: Relapse (OUD)	Cannabis use at baseline was not associated with time to opioid relapse over 12 months (adjusted HR 1.03, 95% CI 0.78–1.36, *p* = 0.84), whereas injection drug use significantly increased relapse risk (HR 2.61, p < 0.001).
Suzuki et al. [106]	Single-arm open-label pilot	5 (20%)	37.8 (7.8)	CBD, 600 mg OD, oral	Beneficial: Craving (OUD)	In buprenorphine-maintained OUD patients, 3 days of CBD resulted in a significant reduction in cue-induced opioid craving (score difference 3.2 at baseline vs. 0.4 post-CBD; *p* = 0.0046).
De Aquino et al. [107]	RCT, double-blind, placebo-controlled crossover	25 (24%)	47.4 (12.3)	Dronabinol, 10–20 mg, oral	No significant effect: Withdrawal severity (OUD)	NS alteration to subjective opioid withdrawal scores vs. placebo during a 5-hour session where the morning methadone dose was withheld.
Elkrief et al. [108]	Prospective cohort (secondary analysis of RCT)	266 (34.6%)	38.9 (10.5)	NA	No significant effect: Consumption (OUD) No significant effect: Craving (OUD) No significant effect: Withdrawal severity (OUD)	Past-week cannabis use was NS associated with consumption (past-week opioid use days; β = −0.06; *p* = 0.15), opioid craving scores (β = −0.05; *p* = 0.49), or withdrawal severity (β = 0.09; *p* = 0.36); Bayes factors consistently supported the null hypothesis (BF < 0.3).
Karoly et al. [109]	RCT, double-blind, placebo-controlled crossover	36 (52.8%)	26.5 (6.8)	CBD, 30–200 mg, oral	No significant effect: Craving (AUD)	CBD administered prior to alcohol did not significantly alter the trajectory of alcohol craving (AUQ) during the descending limb of the blood alcohol curve vs. placebo (Bayesian CIs crossed zero)
Lake et al. [110]	Prospective cohort study	1389 (41%)	41.4 (34.6–47.9)^b^	NA	Beneficial: Consumption (OUD)	Daily cannabis use significantly buffered the risk of daily opioid use associated with subtherapeutic methadone doses (<90 mg/day) (Interaction *p* = 0.010). Low methadone dose increased odds of daily opioid use by 86% (AOR 1.86) among non-daily cannabis users, compared to only 30% (AOR 1.30) among daily cannabis users.
Reddon et al. [111]	Cross-sectional study	205 (32.7%)	39.9 (29.4–53.5)^b^	NA	Beneficial: Craving (OUD) Beneficial: Consumption (OUD)	44.4% used cannabis to manage cravings. This was significantly associated with self-reported reductions in opioid use (aOR 2.13, 95% CI 1.07–4.27) in females (aOR 8.19, 95% CI 1.20–55.81) but not males in gender-stratified analyses. Consumption (OUD): 57.6% reduced opioid use. Associations with daily cannabis use (aOR 3.87, 95% CI 1.16–12.88), significant among participants with moderate–severe pain (aOR 4.44, 95% CI 1.52–12.97).
Suzuki et al. [112]	RCT, double-blind, placebo-controlled crossover	10 (50%)	45.1 (9.1)	Epidiolex, 600 mg, oral	Beneficial: Craving (OUD) No significant effect: Withdrawal severity (OUD)	CBD significantly reduced cue-induced craving vs. placebo in OUD patients under treatment. Post-drug-cue craving was 0.9 (SD 1.1) with CBD vs. 2.4 (SD 1.7) with placebo (*p* = 0.0046). Cue-induced craving (difference score) was 0.2 vs. 1.3 (*p* = 0.040). NS difference between CBD and placebo for withdrawal symptoms (COWS).
Bekier et al. [113]	Cross-sectional survey	118 (28.8%)	42.5 (7.53)	Various, 1.3 g/day, inhaled, oral	Beneficial: Craving (OUD) Beneficial: Consumption (OUD)	68.7% (46/67) used cannabis to cope with craving. 44.8% (30/67) used it to substitute for heroin, rating its effectiveness as 2.7 ± 1.49 (on a school grade scale of 1 = very good to 6 = insufficient; *n* = 48).
Kudrich et al. [114]	Cross-sectional convenience survey	550 (28%)	51.9 (12.6)	Various, various, various	Beneficial: Withdrawal severity (OUD) Beneficial: Craving (OUD)	41.9% used CBD to ease opioid withdrawal symptoms. Of those, 79.7% agreed or strongly agreed that CBD helped. 17.1% reported using CBD specifically to “control their addiction.”
Lake et al. [115]	Prospective cohort	829 (41.4%)	42.4 (35.0–49.2)^b^	NA	No significant effect: Retention (OUD)	Daily cannabis use (vs. < daily) was not associated with treatment discontinuation at 6 months (AOR 0.98; 95% CI 0.66–1.45) or > 6 months (HR 0.91; 95% CI 0.71–1.18). It did not modify the association between low methadone dose and dropout (interaction p > 0.05).
Reddon et al. [116]	Cross-sectional study	297 (31.3%)	44.3 (30.2–54.9)^b^	Various, NA	No significant effect: Consumption (CoUD) Beneficial: Craving (MUD) Beneficial: Consumption (MUD)	45.1% used cannabis to manage stimulant cravings. Among these, 77.6% decreased stimulant use. Among daily crystal methamphetamine users, cannabis use to manage cravings was significantly associated with self-reported reductions in stimulant use (aOR 0.08; 95% CI 0.02–0.37). However, there was NS association with reductions in cocaine/crack users (aOR 0.33; 95% CI 0.04–2.86).
Wolkowicz et al. [117]	RCT, double-blind, placebo-controlled crossover	27 (25.9%)	47.59 (12.03)	Dronabinol, 10–20 mg, oral	Mixed/Partial: Craving (OUD)	NS effect on opioid attentional bias (a cognitive proxy for craving) vs. placebo. However, in the subgroup receiving high-dose methadone (≥90 mg/day), effects were bidirectional: 10 mg THC increased attentional bias, 20 mg decreased it (*p* = 0.002). In the low-dose methadone subgroup (<90 mg/day), it increased attentional bias variability (*p* = 0.029). No effects were observed for pain-related attentional bias.
Hurzeler et al. [118]	RCT, double-blind, placebo-controlled crossover	19 (68.4%)	29.79 (12.59)	CBD, 800 mg/day, oral	Mixed/Partial: Craving (AUD)	CBD significantly reduced self-reported anxiety during alcohol cue exposure (*p* = 0.027), reduced craving (VAS) during the recovery period following cue exposure vs. placebo (*p* = 0.025). However, craving scores during the cue exposure itself did not differ significantly (*p* = 0.437). Higher parasympathetic activity (HF-HRV) across the task (p < 0.001) without impairing cognition or increasing sedation.
Hurzeler et al. [119]	RCT, double-blind, placebo-controlled crossover	18 (72.2%)	29.39 (12.83) range 18–62	CBD, 800 mg/day, oral	No significant effect: Craving (AUD)	NS effect on subjective alcohol craving. NS differences in AUQ scores (*p* = 0.40 pre-scan, *p* = 0.49 post-scan) or VAS craving scores during cue exposure (*p* = 0.728 for treatment interaction). While fMRI revealed that CBD significantly attenuated activation in the precuneus vs. placebo (pFWE = 0.038), this effect was independent of cue type (observed for both alcohol and control cues) and did not translate to reduced craving.
Kirkland et al. [120]	RCT, double-blind, placebo-controlled crossover	36 (69.4%)	20.47 (1.46) range 17.59–22.83	Epidiolex, 600 mg, oral	No significant effect: Craving (AUD) No significant effect: Consumption (AUD)	NS effect on craving during an olfactory alcohol cue-reactivity task (AUQ scores; no significant medication or cue-by-medication interaction). NS difference in the daily drinks consumed in the 7 days following CBD vs. placebo administration (*p* = 0.261). No effect on neural alcohol cue-reactivity (fMRI), dACC neurometabolite levels (Glx/GABA), or physiological reactivity (HRV, SCR).
Mueller et al. [121]	RCT, double-blind, placebo-controlled parallel-group	44 (72.7%)	36.09 (3.27)	CBD, ~150 mg/day, oral	Beneficial: Craving (AUD) No significant effect: Consumption (AUD)	fsCBD (<0.3% THC) significantly reduced craving (PACS) vs. placebo at week 8 (*p* = 0.014) and week 16 (p < 0.001). bsCBD (no THC) did NS reduce craving vs. placebo. NS differences between groups (fsCBD, bsCBD, placebo) in number of drinks per drinking day (TLFB) or alcohol dependence severity (ADS) during the treatment period.
Shulman et al. [122]	Prospective cohort (secondary analysis of RCT)	569 (29.7%)	34 (NA)	NA	No significant effect: Abstinence (OUD) No significant effect: Relapse (OUD)	Cross-lagged mediation models found NS effect of cannabis use on subsequent opioid use over 24 weeks. Cannabis use at earlier timepoints did not predict opioid use at later timepoints, nor did it mediate treatment outcomes.
Zimmermann et al. [123]	RCT, double-blind, placebo-controlled parallel-group	28 (32.1%)	35.8 (12.1)	CBD, 800 mg, oral	Beneficial: Craving (AUD)	Following a combined stress-and-alcohol-cue exposure, the placebo group showed an increase in craving (AUQ score ↑ ~15 points), while the CBD group showed a blunted response (↑ ~5 points) (*p* = 0.025 for interaction). In a subsequent fMRI task, the CBD group showed lower cue-induced craving (*p* = 0.015, n2 = 0.23$) and activation in the nucleus accumbens (p < 0.001). Higher CBD plasma levels correlated with lower craving (*r* = −0.394, pFDR = 0.030).

Abbreviations: SUD, substance use disorder; OUD, opioid use disorder; AUD, alcohol use disorder; CoUD, cocaine use disorder; TUD, tobacco use disorder; MUD, methamphetamine use disorder. NA, not available. NS, not significant. HR, hazard ratio. OR, odds ratio.

^a^ *Calculated on 176 first admission patients, excluding 20 re-entering.*

^b^ *Median (IQR)*

Of the 17 RCTs, 12 were rated low risk of bias on RoB 2, 4 raised some concerns, and 1 was rated high risk (eFigures 1a–h). Among the 47 cohort studies assessed with ROBINS-I, 11 were rated low risk, 27 moderate, 7 serious, and 2 critical, with bias due to confounding the most frequently flagged domain. Of the 33 studies assessed with JBI checklists (20 cross-sectional, 4 case series, 9 qualitative), 21 were rated as low concern, 11 as moderate concern, and 1 as high concern.

**Figure 2.**
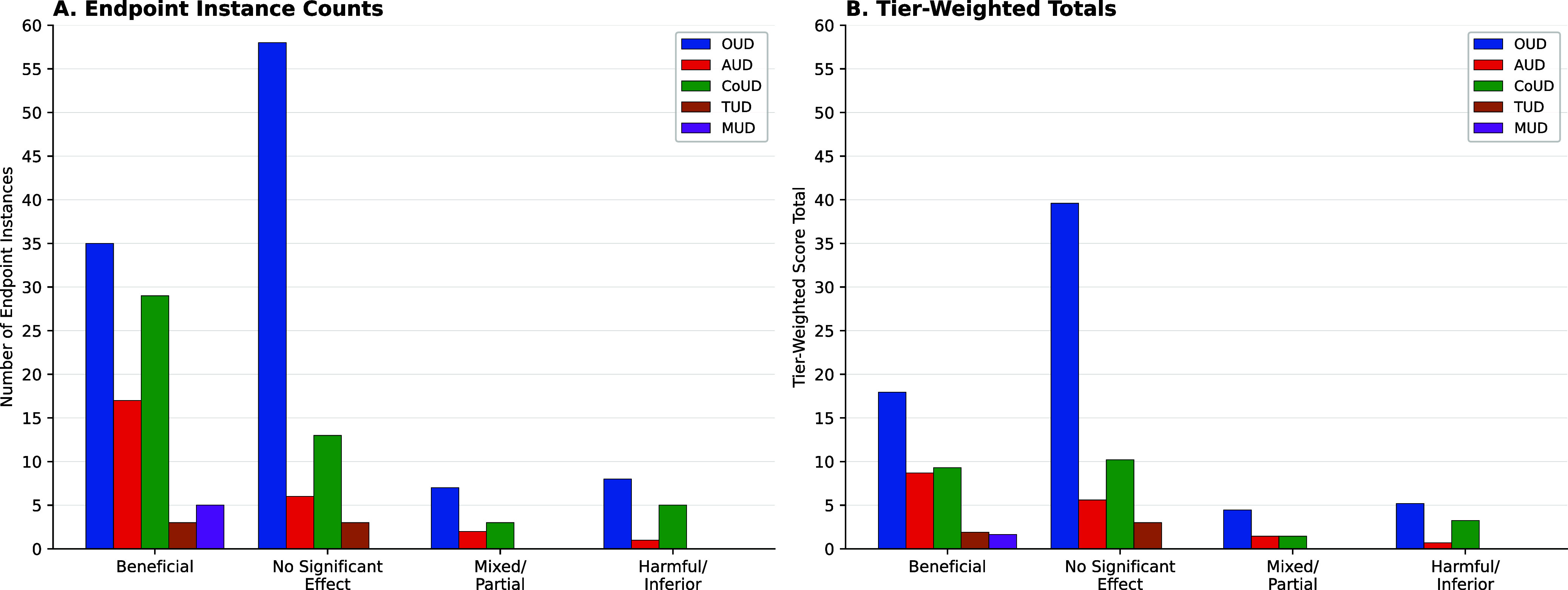
SUD endpoint instance counts and tier-weighted totals by direction-of-effect category and substance use disorder. Panel A shows raw endpoint instance counts; Panel B shows tier-weighted score totals. SUD indicates substance use disorder; OUD, opioid use disorder; AUD, alcohol use disorder; CoUD, cocaine use disorder; TUD, tobacco use disorder; MUD, methamphetamine use disorder. Figure 2. long description.

Opioid use disorder (OUD) dominated the evidence base, contributing 108 of 195 endpoint instances (55.4%), followed by cocaine use disorder (CoUD; 50, 25.6%), alcohol use disorder (AUD; 26, 13.3%), tobacco use disorder (TUD; 6, 3.1%), and methamphetamine use disorder (MUD; 5, 2.6%). Nine studies contributed to more than one SUD, and 49 contributed to more than one endpoint instance. The distribution of direction-of-effect categories varied markedly by disorder (Figure 2; Table 2). Most No Significant Effect instances arose in OUD (58/80; 72.5%), and OUD also accounted for the largest share of Harmful/Inferior instances (8/14; 57.1%). In contrast, for all other SUDs, Beneficial classifications outnumbered non-Beneficial ones, predominantly accounted for by short-term SUD endpoints craving, withdrawal severity, and consumption. For MUD, all five instances were Beneficial, though numbers were small. Expressed as a percentage of each disorder’s total endpoint instances, the direction-of-effect distributions were directly comparable across disorders (full distributions in Table 2). No Significant Effect predominated only in OUD, at 53.7% of its instances, against 23.1% in AUD and 26.0% in CoUD. Beneficial classifications represented 65.4% of AUD, 58.0% of CoUD, and all five MUD instances, compared with 32.4% in OUD, while TUD was evenly split at 50.0%.

**Table 2.** SUD endpoint instances and tier score totals, with breakdown per direction-of-effect category and SUD endpoints for each SUD Table 2. long description.

	OUD		AUD		CoUD		TUD		MUD	
	*n*	ΣTier	*n*	ΣTier	*n*	ΣTier	*n*	ΣTier	*n*	ΣTier
**Beneficial**										
Retention	2	1.3	0	0	0	0	0	0	0	0
Relapse	2	0.7	2	0.7	4	1.2	0	0	0	0
Abstinence	4	1.75	2	0.7	4	1.2	1	0.45	0	0
Craving	11	6	4	2.7	10	2.8	0	0	2	0.7
Withdrawal	9	5	4	1.8	7	2.25	0	0	1	0.25
Consumption	7	3.2	5	2.8	4	1.85	2	1.45	2	0.7
Total	35	17.95	17	8.7	29	9.3	3	1.9	5	1.65
**No significant effect**										
Retention	20	13.6	0	0	1	0.7	0	0	0	0
Relapse	6	4.1	0	0	3	2.4	0	0	0	0
Abstinence	13	8.3	1	0.6	2	1.2	0	0	0	0
Craving	6	4.25	3	3	3	2.45	2	2	0	0
Withdrawal	8	5.95	0	0	2	2	1	1	0	0
Consumption	5	3.4	2	2	2	1.45	0	0	0	0
Total	58	39.6	6	5.6	13	10.2	3	3	0	0
**Mixed/Partial**										
Retention	1	0.7	0	0	1	0.6	0	0	0	0
Relapse	1	0.45	0	0	0	0	0	0	0	0
Abstinence	2	1.4	0	0	1	0.6	0	0	0	0
Craving	1	1	1	1	1	0.25	0	0	0	0
Withdrawal	2	0.9	0	0	0	0	0	0	0	0
Consumption	0	0	1	0.45	0	0	0	0	0	0
Total	7	4.45	2	1.45	3	1.45	0	0	0	0
**Harmful/Inferior**										
Retention	4	2.5	0	0	0	0	0	0	0	0
Relapse	3	2	1	0.7	1	0.7	0	0	0	0
Abstinence	1	0.7	0	0	0	0	0	0	0	0
Craving	0	0	0	0	2	1.15	0	0	0	0
Withdrawal	0	0	0	0	2	1.4	0	0	0	0
Consumption	0	0	0	0	0	0	0	0	0	0
Total	8	5.2	1	0.7	5	3.25	0	0	0	0

Abbreviations: n, number of SUD endpoint instances; ΣTier, tier score total. Tier weights: RCT (1.00), prospective cohort (0.70), retrospective cohort (0.60), cross-sectional (0.45), case series (0.35), qualitative (0.25).

Abbreviations: OUD, opioid use disorder; AUD, alcohol use disorder; CoUD, cocaine use disorder; TUD, tobacco use disorder; MUD, methamphetamine use disorder.

Tier-weighted totals (Figure 2B) recalibrated the apparent distribution of findings. In OUD, the Beneficial tier-weighted sum (17.95) was substantially lower than the No Significant Effect sum (39.60), because the Beneficial signal derived partly from lower-tier designs whose contributions were proportionally reduced. The same pattern was visible across all SUDs when raw counts (Figure 2A) were compared with tier-weighted totals (Figure 2B). In AUD, however, the Beneficial sum (8.70) still exceeded No Significant Effect (5.60) after weighting, suggesting a more robust symptomatic signal. In CoUD, the balance shifted. Raw Beneficial instances outnumbered No Significant Effect (29 vs. 13), but tier-weighted totals reversed this (9.30 vs. 10.20), indicating that much of the CoUD Beneficial signal came from lower-evidence designs. TUD (1.90 Beneficial, 3.00 No Significant Effect) and MUD (1.65 Beneficial, all weighted evidence) contributed few but directionally notable instances.

**Figure 3.**
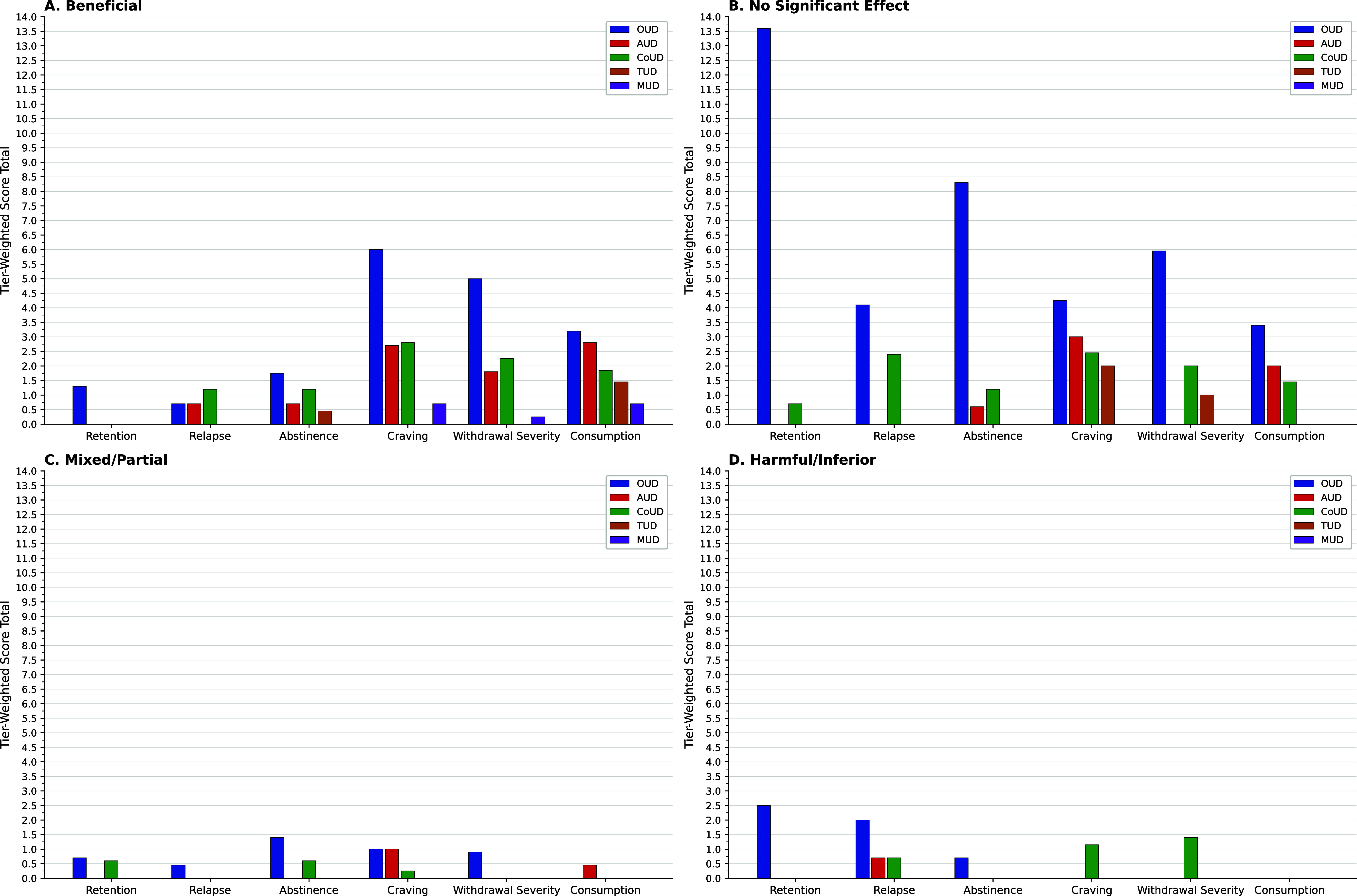
Tier-weighted SUD endpoint total scores grouped by direction-of-effect category (A, Beneficial; B, No Significant Effect; C, Mixed/Partial; D, Harmful/Inferior), color-coded by substance use disorder. Figure 3. long description.

The 195 SUD endpoint instances comprised 29 retention, 23 relapse, 31 abstinence, 46 craving, 36 withdrawal severity, and 30 consumption. Two patterns were consistent across SUDs (Table 2; Figure 3). Patient-reported symptom endpoints, craving and withdrawal severity, displayed the highest Beneficial counts, with craving contributing 46 instances and withdrawal severity 36, the majority classified Beneficial. The highest Beneficial peaks arose from craving, withdrawal severity, and consumption in OUD, AUD, and CoUD. In contrast, retention, relapse, and abstinence aggregated in non-Beneficial categories. Within OUD, retention showed marked No Significant Effect dominance (Beneficial: 2, 1.30; No Significant Effect: 20, 13.60; Mixed/Partial: 1, 0.70; Harmful/Inferior: 4, 2.50). Abstinence (Beneficial: 4, 1.75; No Significant Effect: 13, 8.30; Mixed/Partial: 2, 1.40; Harmful/Inferior: 1, 0.70) and relapse (Beneficial: 2, 0.70; No Significant Effect: 6, 4.10; Mixed/Partial: 1, 0.45; Harmful/Inferior: 3, 2.00) demonstrated the same pattern. Unlike in OUD, CoUD, and TUD, cannabinoid exposure studies in AUDs presented a higher tier total score for Beneficial (8.70) than for No Significant Effect (5.60). In CoUD, SUD endpoints were higher by instance count in the Beneficial category, 29, however, when considering tier scores, the No Significant Effect direction-of-effect registered the highest total value (10.20).

**Figure 4.**
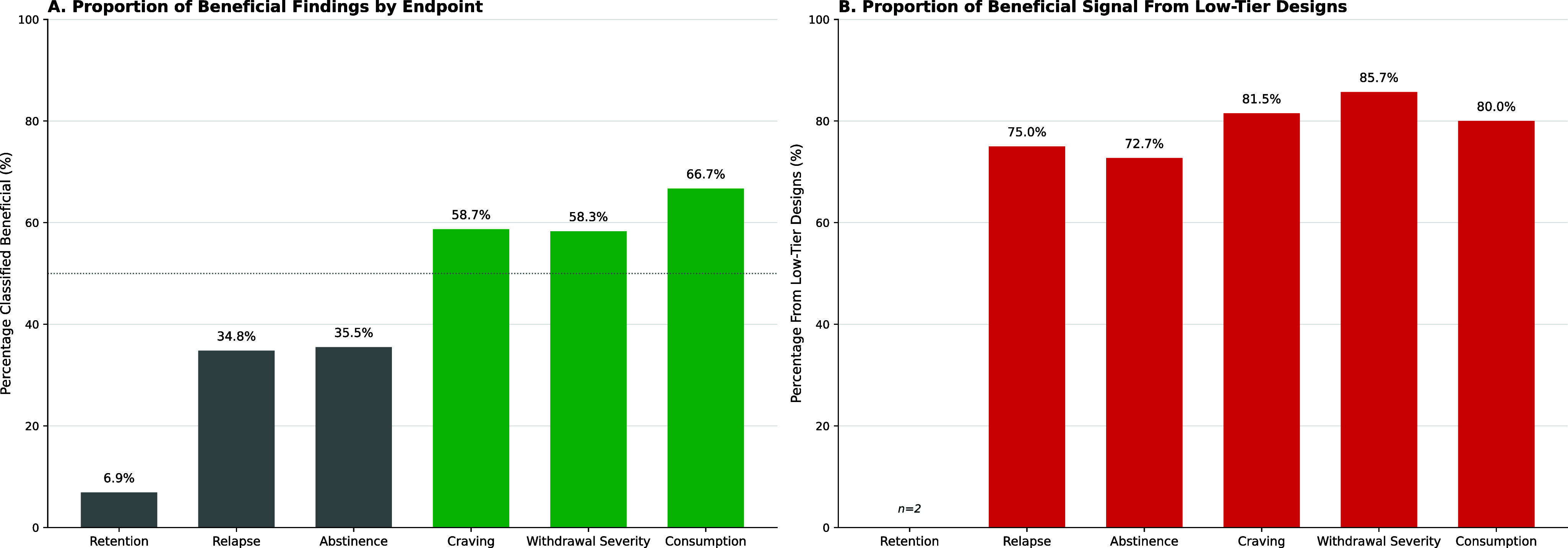
Evidence quality gradient across SUD endpoints. Panel A shows the proportion of endpoint instances classified as Beneficial for each of the six prespecified endpoints, with bars colored green where most instances were Beneficial (above the 50% reference line) and gray where fewer than half were Beneficial. Panel B shows the proportion of those Beneficial findings derived from lower-tier designs (cross-sectional, case series, and qualitative), shown in red. Retention is based on only two Beneficial instances (*n* = 2). Figure 4. long description.

Across designs, outcome verification varied from objective measures to self-report. Objective verification, using biochemical testing or administrative records, was most common for the three sustained outcome endpoints, namely retention, relapse, and abstinence. For each of these SUD endpoints, the majority of instance counts were classified in non-Beneficial direction-of-effect categories (retention: 27/29 [93.1%]; relapse: 15/23 [65.2%]; abstinence: 20/31 [64.5%]) (Table 2), and nearly all of these non-Beneficial findings were extracted from higher-tier designs, including RCTs, prospective and retrospective studies (retention: 27/27 [100%]; relapse: 14/15 [93.3%]; abstinence: 18/20 [90.0%]) (Table 1). The inverse is noticeable for shorter-term SUD endpoints that deal with a more immediate context post-intervention. A higher percentage of studies for craving, withdrawal severity, and consumption report SUD endpoint instance counts that cluster in the Beneficial category (craving: 27/46 [58.7%]; withdrawal severity: 21/36 [58.3%]; consumption: 20/30 [66.7%]), but the overwhelming majority are derived from low-evidence studies, including qualitative, cross-sectional, and case studies (craving: 22/27 [81.5%]; withdrawal severity: 18/21 [85.7%]; consumption: 16/20 [80.0%]). This inverse relationship between endpoint horizon and evidence tier is a defining feature of the data set (Figure 4). The outcomes for which cannabinoids appear most consistently beneficial are precisely those supported by the weakest designs, while the outcomes most relevant to sustained recovery are evaluated by stronger designs that predominantly find no effect.

## Discussion

This review set out to determine whether cannabis or specific cannabinoid formulations confer clinically meaningful benefit across opioid, alcohol, cocaine, tobacco, and methamphetamine use disorders, classifying studies by design quality and organizing findings by endpoint so that stronger designs and sustained outcomes carried the greatest weight in the conclusions. Against that aim, the central finding is a consistent divergence between symptomatic and sustained therapeutic outcomes of cannabinoid exposure across SUDs. Short-term endpoints (craving, withdrawal severity, and consumption) cluster in the Beneficial direction with comparatively higher tier-weighted totals, while long-term endpoints (retention, relapse, and abstinence) predominantly populate the No Significant Effect category, most strikingly in OUD (Figures 2–4; Table 2). This divergence reflects the inverse relationship between endpoint horizon and evidence tier established in the Results, whereby the most consistent benefits attach to the outcomes assessed by the weakest designs. Together, these findings support a cautious interpretation. Cannabinoid strategies may improve short-horizon symptoms but the current evidence base does not demonstrate that such improvement translates into durable changes in treatment trajectory.

In OUD, where the evidence base is largest, the asymmetry between short-term and long-term SUD endpoints is especially clear, with the heaviest No Significant Effect weights falling on retention and abstinence and exceeding the corresponding symptomatic weights (Table 2). Several methadone and clinic cohorts that verified exposure by urinalysis and tracked administrative retention found no association between cannabis use and illicit opioid use or treatment retention once core clinical covariates were included. In these analyses, cannabis coefficients typically attenuated to null or fluctuated inconsistently after adjustment for factors such as opioid agonist dose, baseline severity, and visit adherence [29, 32, 38, 39, 61, 67, 75, 85, 105]. Consistently, across OUD studies, dose, treatment assignment, and engagement emerged as the dominant drivers of retention and abstinence, and the apparent cannabis signal diminished when these determinants were modeled explicitly [42, 54, 57, 66, 71, 86, 122]. Relapse in OUD demonstrated a similar pattern, with the highest registered tier scores in the No Significant Effect [38, 40, 63, 104, 105, 122] and Harmful/Inferior [33, 53, 77] direction-of-effect categories, with a smaller Beneficial component [58, 70].

AUD and CoUD display related but not identical patterns. In AUD, Beneficial tier weight is concentrated in craving [58, 70, 121, 123], consumption [44, 48, 89, 96, 100], and withdrawal severity [44, 48, 58, 100]. No Significant Effect appears most in craving [109, 119, 120] and consumption [120, 121] within AUD, while harmful signals are scarce [40]. In CoUD, the Beneficial signal again concentrates in short-term SUD endpoints and derives predominantly from qualitative, cross-sectional, and case study designs rather than controlled ones (Table 2), with smaller low-evidence contributions to relapse and abstinence. Qualitative and field investigations consistently describe intentional, post-crack cannabis use to manage the “come down,” reducing agitation, dysphoria, and urges across multiple settings. In mixed methods and longitudinal work, reductions in crack use are sometimes observed in the period after these cannabis substitution episodes rather than during use, suggesting a temporal asymmetry that is mechanistically plausible and clinically testable. This pattern warrants targeted prospective designs that anchor exposure, symptom trajectories, and subsequent consumption temporally to determine whether post-episode mitigation translates into reproducible reductions in stimulant use.

Cannabinoid exposure in CoUD does exhibit a notable non-beneficial footprint. No Significant Effect totals are distributed across all six SUD endpoints [21, 38, 40, 50, 90, 98, 101, 116], while the Harmful/Inferior category includes SUD endpoints in specific observational contexts [65, 80, 95]. Inpatient detox cohorts indicate a correlation between earlier initiation of cannabis and long-duration or heavy use with higher withdrawal scores, more intense craving, and increased re-hospitalizations during CoUD detox, delineating an adverse clinical phenotype within that context [65, 95]. This inpatient signal coexists with the community “come down” narrative, implying that setting, exposure history, and severity shape directionality in CoUD. Future studies should stratify by age of cannabis onset and cumulative exposure to clarify when cannabis use is palliative versus prognostically unfavorable. TUD and MUD contribute far fewer instances overall. Signals trend Beneficial mostly for craving, withdrawal severity, and consumption, with fewer non-beneficial weight recorded [59, 84, 89, 94, 116], but numbers are small and should be read as preliminary.

Taken together, these disorder-specific patterns converge on a single overarching observation. Although cannabinoids may reduce uncomfortable or motivating symptoms in the near term, there is little consistent evidence that such modulation translates into improved treatment course or attainment of sustained non-use. The relative consistency of Beneficial symptom signals suggests that cannabinoids may have a role as adjuncts for short-horizon symptom targets, reducing distressing cravings, mitigating withdrawal experiences, and diminishing overall short-term consumption, at times when such effects can meaningfully support patient engagement or comfort. Such short-term relief from craving, withdrawal, and consumption may itself be valued by patients, even where it does not translate into longer-term treatment benefit. Conversely, the preponderance of No Significant Effect for sustained SUD endpoints, especially in OUD, argues against extrapolating those short-term benefits into assumptions about enduring treatment success. Within the scope of this review, symptom relief is not shown to bridge into sustained therapeutic trajectory or verified non-use at scale.

The tier-weighted framework explains why the short-term/long-term divergence holds even when unweighted counts appear encouraging. By assigning greater weight to randomized and prospective designs, it shows that sustained outcomes are evaluated mainly in higher-tier studies clustering in No Significant Effect, while symptomatic benefits rest disproportionately on lower-tier designs. The few low-tier signals of mild gain in sustained outcomes do not subvert this trend.

This concentration of benefit in the weaker designs is the review’s second principal message, and it carries a specific interpretive risk. Most lower-tier studies are open-label, observational, or qualitative and lack the blinding and placebo control needed to separate a pharmacological effect from expectancy. Cannabinoids are known to produce substantial placebo and expectancy responses. A meta-analysis of double-blind randomized trials in clinical pain found that placebo produced pain reduction comparable to the active cannabinoid [124], and balanced placebo experiments show that the expectancy of having received THC alters subjective and behavioral responses independently of the drug itself [125]. Because craving and withdrawal severity, the endpoints where benefit is most consistent, are largely self-reported and particularly sensitive to expectancy, the symptomatic gains seen in uncontrolled and lower-tier studies may partly reflect these mechanisms rather than a specific cannabinoid action. Caution is, therefore, warranted in attributing even short-term symptom relief to cannabinoids until it is confirmed in adequately powered, placebo-controlled, blinded trials.

A cautious, adjunctive stance appears warranted. The review’s results speak to clinical expectations rather than to the specifics of how cannabinoids should be administered. Where cannabinoids are considered, they are best framed as additions to evidence-based care directed at explicit short-term symptom targets, the only domain in which benefit was observed, and not as a means of improving retention, abstinence, or relapse prevention, for which the present literature shows no consistent effect, particularly in OUD. The non-beneficial and occasionally harmful signals seen in some observational contexts warrant monitoring rather than a presumption of safety. For services and policy makers, these results argue against blanket endorsement while leaving room for methodologically structured exploration where symptom burdens are high and conventional options are constrained.

These findings extend and sharpen themes identified by prior disorder-specific and cannabinoid-specific reviews. In OUD, previous syntheses have reported reductions in craving and anxiety [126], benefit in withdrawal settings on nausea and muscle spasms [127], and modest opioid withdrawal alleviation by dronabinol within a narrow dose range [128]. CBD has been characterized as showing some indication of benefit on opioid craving and anxiety [129], and a cross-SUD review reported beneficial CBD effects in TUD and OUD [130]. Mechanistic syntheses have invoked CBD effects on stress reactivity, sleep, and cue processing to justify adjunctive use [131]. However, when attention shifts from proximal symptoms to sustained outcomes, prior evidence becomes more neutral. Meta-analysis of longitudinal OUD data found no significant cannabis association with mitigating non-medical opioid use [132], and skepticism persists regarding cannabinoid efficacy on stimulant use outcomes [130, 133, 134]. Our tiered synthesis renders those differences visible by disaggregating endpoints and weighting designs within the largest evidence base yet considered, clarifying why mechanistic plausibility should not be conflated with clinical durability.

This review’s principal strengths are its cross-disorder scope and systematic coverage, yielding the largest eligible corpus of human studies on cannabinoid exposure across five SUDs. Careful SUD endpoint mapping upholds the distinction of patient-oriented symptom experience from longer-horizon clinical outcomes and verified use. The tier-weighted scheme privileges controlled designs while incorporating observational and lived-experience evidence, permitting a wide-angle view of efficacy while guarding against overinterpretation of acute laboratory signals.

Several limitations warrant consideration. The corpus remains heterogeneous across exposure definitions, time horizons, outcome definitions, and measurement methods. Many studies rely on self-report for exposure and sometimes for outcomes, complicating dose–response inference. Objective verification, when present, is most common for outcomes such as administrative retention or biochemically verified use, but even then, follow-up durations and analytic choices vary. Observational designs frequently face residual confounding, including time-varying co-use and symptom severity, and selection mechanisms that can make cannabis exposure a marker of underlying risk rather than a modifiable driver of outcomes. Few randomized trials have sufficient size or duration to test whether acute symptom relief mediates clinically meaningful change over the months in which relapse and sustained non-use are decided. Publication bias is plausible, particularly for early-phase symptom results, and may overstate the apparent consistency of Beneficial findings for craving and withdrawal severity. Representativeness is uneven. Opioid medication programs figure prominently in the OUD evidence, while alcohol and cocaine cohorts often come from inpatient or post-discharge settings. Tobacco and methamphetamine domains are comparatively sparse and geographically constrained. In addition, the tier-weighting framework was compiled by the authors for this review and has not been independently validated, so the tier-weighted values should be read as ordinal scaling aids rather than as validated quantitative estimates.

Future research should close three gaps. First, adequately powered, longer-follow-up RCTs that integrate cannabinoids as adjuncts to standard care, with biochemically verified outcomes, time-horizon stratification to map short-term symptom change onto medium-term outcomes, clear specification of product class, dose, route, the cannabidiol (CBD) and tetrahydrocannabinol (THC) ratio (CBD:THC ratio), and systematic monitoring of adverse effects. These trials should be placebo-controlled and blinded, and given the prominence of expectancy in cannabinoid research, future work should also characterize the placebo and expectancy contribution to the SUD-relevant outcomes reported here, across both symptomatic and sustained endpoints, for example, through balanced placebo designs that separate pharmacological from expectancy effects. Second, real-world studies should embrace target-trial emulation, time-varying confounding control, and transparent exposure characterization, acknowledging policy heterogeneity and product variability that plausibly moderate effects. Third, a harmonized endpoint framework, adopted prospectively across disorders, would improve comparability and facilitate the kind of cross-SUD synthesis attempted here.

When disorder, endpoint, and design are considered together, the evidence from 97 studies encompassing 41,954 participants supports a selective conclusion. Cannabinoid strategies most consistently favor symptom relief, targeting craving, withdrawal severity, and reduction of consumption in the acute treatment stage. The firmest conclusions concern OUD, where the eligible literature is by far the most extensive and cannabinoids are predominantly ineffective for enduring clinical outcomes such as retention, relapse, and abstinence. For AUD, CoUD, TUD, and MUD, far fewer studies met eligibility, with TUD and MUD represented by only a handful of instances, so the findings there, in either direction, should be read as preliminary rather than definitive. This disparity reflects the state of the published evidence rather than any restriction of scope, since all studies meeting the prespecified criteria were included. Within the scope and limits of this review, cannabinoids might be considered for short-horizon symptom targets under protocolized conditions, recognizing that even this short-term benefit rests largely on weaker designs, but they should not be assumed to improve long-term treatment trajectory without further evidence. This interpretation integrates the strengths of a tier-aware synthesis with the discipline of endpoint-specific reporting and is offered to inform clinical decision making, service planning, and the design of next-generation trials aligned to sustained therapeutic outcomes.

## Supporting information

10.1192/j.eurpsy.2026.12236.sm001 Zammit Dimech et al. supplementary material Zammit Dimech et al. supplementary material

## Data Availability

Data extracted and synthesized for this review are reported in the manuscript, tables, and supplementary material. Any additional data can be made available by the corresponding author upon reasonable request.

## Supplementary material

The supplementary material for this article can be found at http://doi.org/10.1192/j.eurpsy.2026.12236.

## Long descriptions

### Figure 1. Long description

The flowchart is organized into four vertical stages labeled on the left.

1. Identification: The top box shows 11,134 records identified from two databases: PubMed (n=4539) and Embase (n=6595). An arrow points right to a box indicating 1,758 duplicate records were excluded. A downward arrow leads to a box with 9,376 records remaining after duplicate removal.

2. Screening: A downward arrow leads to a box showing 9,376 records screened. An arrow points right to a box indicating 9,252 records were excluded at the title or abstract level.

3. Eligibility: A downward arrow leads to a box showing 124 records assessed for eligibility via full text. An arrow points right to a box listing 27 records excluded for specific reasons: 7 for mechanistic or cognitive measures with no clinical S U D endpoint, 6 for use of C B 1 antagonists or inverse agonists, 6 for no S U D endpoint recorded for analysis, 3 for no cannabis exposure analyzed, 4 for healthy volunteers, and 1 for polydrug exposure.

4. Inclusion: A final downward arrow leads to the bottom box indicating 97 studies were included in the systematic review.

Navigate back to Figure 1..

### Table 1. Long description

The table is organized into seven columns: Study, Study design, Sample size (female percentage), Mean age (S D) in years, Form, dose, route, Direction-of-effect: S U D endpoint (S U D), and Outcome.

Key entries include:

* Gossop et al. [28]: Cross-sectional retrospective survey of 50 participants (30% female). Outcome: Mixed/Partial effect on withdrawal severity in O U D. 22 participants used cannabis; 12 reported it made withdrawal worse, 6 said it reduced distress.

* Saxon et al. [29]: Retrospective analysis of 98 participants (0% female). Outcome: No significant effect on abstinence or retention in O U D.

* Wasserman et al. [33]: Prospective observational study of 74 participants (40.5% female). Outcome: Harmful/Inferior effect on relapse in O U D. Baseline cannabis use significantly increased heroin lapse risk.

* Best et al. [34]: Cross-sectional survey of 200 participants (30% female). Outcome: Beneficial effect on consumption in O U D. Daily users reported fewer days of heroin use.

* Labigalini et al. [35]: Prospective case series of 25 participants (0% female). Outcome: Beneficial effect on abstinence, craving, and withdrawal in Co U D. 68% ceased crack use.

* Dreher [37]: Qualitative ethnographic study of 33 females (100%). Outcome: Beneficial effect on craving, relapse, and abstinence in Co U D. 92.8% attributed stopping crack use to ganja.

* Morgan et al. [59]: R C T pilot of 24 participants (50% female) using C B D inhalers. Outcome: Beneficial effect on consumption in T U D. C B D group reduced cigarettes by approximately 40%.

* Hurd et al. [88]: R C T of 42 participants (16.7% female) using Epidiolex. Outcome: Beneficial effect on craving in O U D. Lower cue-induced craving persisting 7 days post-dose.

* Lake et al. [110]: Prospective cohort of 1389 participants (41% female). Outcome: Beneficial effect on consumption in O U D. Daily cannabis use buffered the risk of daily opioid use associated with low methadone doses.

Abbreviations used: S U D (substance use disorder), O U D (opioid use disorder), A U D (alcohol use disorder), Co U D (cocaine use disorder), T U D (tobacco use disorder), M U D (methamphetamine use disorder), N A (not available), N S (not significant).

Navigate back to Table 1..

### Figure 2. Long description

Panel A, titled Endpoint Instance Counts, has a y-axis labeled Number of Endpoint Instances ranging from 0 to 60. The x-axis lists four categories: Beneficial, No Significant Effect, Mixed forward slash Partial, and Harmful forward slash Inferior. Each category contains a cluster of five colored bars representing O U D in blue, A U D in red, Co U D in green, T U D in orange, and M U D in purple. In the Beneficial category, O U D is highest at 35, followed by Co U D at 29. In the No Significant Effect category, O U D peaks at 58.

Panel B, titled Tier-Weighted Totals, has a y-axis labeled Tier-Weighted Score Total ranging from 0 to 60. The x-axis categories and legend remain the same as Panel A. The overall trends are similar but the magnitudes are lower. In the Beneficial category, O U D is approximately 18, while Co U D and A U D are around 9. In the No Significant Effect category, O U D remains the highest peak at approximately 40. For both panels, T U D and M U D consistently show the lowest counts and scores across all effect categories.

Navigate back to Figure 2..

### Table 2. Long description

The table is organized into four primary sections based on direction-of-effect. Each section lists six endpoints: Retention, Relapse, Abstinence, Craving, Withdrawal, and Consumption, followed by a section total.

1. Beneficial Effect Section:

- O U D: Total n = 35, Sigma Tier = 17.95. Highest endpoint is Craving (n = 11, Sigma Tier = 6).

- A U D: Total n = 17, Sigma Tier = 8.7. Highest endpoint is Consumption (n = 5, Sigma Tier = 2.8).

- Co U D: Total n = 29, Sigma Tier = 9.3. Highest endpoint is Craving (n = 10, Sigma Tier = 2.8).

- T U D: Total n = 3, Sigma Tier = 1.9.

- M U D: Total n = 5, Sigma Tier = 1.65.

2. No Significant Effect Section:

- O U D: Total n = 58, Sigma Tier = 39.6. Highest endpoint is Retention (n = 20, Sigma Tier = 13.6).

- A U D: Total n = 6, Sigma Tier = 5.6.

- Co U D: Total n = 13, Sigma Tier = 10.2.

- T U D: Total n = 3, Sigma Tier = 3.

- M U D: Total n = 0, Sigma Tier = 0.

3. Mixed/Partial Effect Section:

- O U D: Total n = 7, Sigma Tier = 4.45.

- A U D: Total n = 2, Sigma Tier = 1.45.

- Co U D: Total n = 3, Sigma Tier = 1.45.

- T U D and M U D: All values are 0.

4. Harmful/Inferior Effect Section:

- O U D: Total n = 8, Sigma Tier = 5.2.

- A U D: Total n = 1, Sigma Tier = 0.7.

- Co U D: Total n = 5, Sigma Tier = 3.25.

- T U D and M U D: All values are 0.

Abbreviations used: O U D (Opioid Use Disorder), A U D (Alcohol Use Disorder), Co U D (Cocaine Use Disorder), T U D (Tobacco Use Disorder), M U D (Methamphetamine Use Disorder).

Navigate back to Table 2..

### Figure 3. Long description

A four-panel set of bar charts labeled A through D. Each chart shares a Y-axis titled Tier-Weighted Score Total ranging from 0.0 to 14.0 in increments of 0.5. The X-axis lists six endpoint categories: Retention, Relapse, Abstinence, Craving, Withdrawal Severity, and Consumption. A legend in each panel identifies five substance use disorders: O U D in blue, A U D in red, C o U D in green, T U D in orange, and M U D in purple.

* Panel A, Beneficial: O U D shows the highest scores in Craving at 6.0 and Withdrawal Severity at 5.0. Other categories remain below 3.5.

* Panel B, No Significant Effect: This panel contains the highest overall values. O U D peaks at 13.5 for Retention and 8.3 for Abstinence. Craving and Withdrawal Severity for O U D are around 4.3 and 6.0 respectively.

* Panel C, Mixed or Partial: All scores are significantly lower, with no bar exceeding 1.5. O U D and A U D show small peaks in Craving and Abstinence.

* Panel D, Harmful or Inferior: Scores are also low, with O U D peaking at 2.5 for Retention and 2.0 for Relapse. C o U D shows values between 1.0 and 1.5 for Craving and Withdrawal Severity.

Navigate back to Figure 3..

### Figure 4. Long description

A two-panel figure analyzing S U D endpoints.

Panel A, titled Proportion of Beneficial Findings by Endpoint, has a y-axis labeled Percentage Classified Beneficial percent ranging from 0 to 100. A horizontal dashed reference line is at 50 percent. Six bars from left to right are:

* Retention: 6.9 percent (gray)

* Relapse: 34.8 percent (gray)

* Abstinence: 35.5 percent (gray)

* Craving: 58.7 percent (green)

* Withdrawal Severity: 58.3 percent (green)

* Consumption: 66.7 percent (green)

Panel B, titled Proportion of Beneficial Signal From Low-Tier Designs, has a y-axis labeled Percentage From Low-Tier Designs percent ranging from 0 to 100. Six bars from left to right are:

* Retention: No bar shown, labeled with n equals 2

* Relapse: 75.0 percent (red)

* Abstinence: 72.7 percent (red)

* Craving: 81.5 percent (red)

* Withdrawal Severity: 85.7 percent (red)

* Consumption: 80.0 percent (red)

Navigate back to Figure 4..
