# Supplementary material for: Cannabinoid exposure across substance use disorders: Short-term symptom benefits without sustained therapeutic gains in a tier-weighted systematic review
Source: Eur Psychiatry. 2026 Jul 17;69(1):e73. doi: 10.1192/j.eurpsy.2026.12236 (PMC13420148; doi:10.1192/j.eurpsy.2026.12236)
Supplement: Zammit Dimech et al. supplementary material [file S0924933826122366sup001.docx]

**Supplement**

**Cannabinoid exposure across substance use disorders: short-term symptom benefits without sustained therapeutic gains in a tier-weighted systematic review**

**eAppendix.** Search Strategies

**eTable 1.** Synthesis Without Meta-Analysis (SWiM) Reporting Checklist

**eTable 2.** Studies Excluded At Full-Text Assessment

**eTable 3.** Study-level Effect Estimates And Confidence Intervals, By Disorder And Endpoint

**eFigures 1a-i.** Risk of Bias Assessments

**eAppendix.** Search Strategies

**PubMed Search Query**

*Platform: PubMed/MEDLINE (NLM). Dates: 1 January 1975 to 30 June 2025. Results: 4,539.*

("Cannabis/therapeutic use"[Mesh] OR "Cannabinoids/therapeutic use"[Mesh] OR

"Tetrahydrocannabinol/therapeutic use"[Mesh] OR "Cannabidiol/therapeutic use"[Mesh] OR

"Dronabinol/therapeutic use"[Mesh] OR "Nabilone/therapeutic use"[Mesh]

OR

(cannabis OR marijuana OR cannabinoid* OR thc OR "delta-9-tetrahydrocannabinol"

OR "Δ9-THC" OR cannabidiol OR cbd OR dronabinol OR nabilone OR nabiximols

OR sativex)[tiab]

AND (treat*[tiab] OR therap*[tiab] OR adjunct*[tiab] OR substitution[tiab]

OR replacement[tiab] OR maintenance[tiab] OR detox*[tiab]

OR "withdrawal management"[tiab] OR craving[tiab]

OR "relapse prevention"[tiab])

)

NOT (rimonabant[tiab] OR SR141716[tiab] OR taranabant[tiab] OR ibipinabant[tiab]

OR surinabant[tiab] OR otenabant[tiab] OR "CB1 antagonist"[tiab]

OR "CB1 receptor antagonist"[tiab] OR "cannabinoid-1 antagonist"[tiab]

OR "inverse agonist*"[tiab])

AND

("Substance-Related Disorders"[Majr] OR "Opioid-Related Disorders"[Majr] OR

"Alcohol-Related Disorders"[Majr] OR "Cocaine-Related Disorders"[Majr] OR

"Amphetamine-Related Disorders"[Majr] OR "Tobacco Use Disorder"[Majr]

OR

(opioid*[tiab] OR heroin[tiab] OR cocaine[tiab] OR crack[tiab]

OR amphetamine*[tiab] OR methamphetamine[tiab] OR stimulant*[tiab]

OR alcohol*[tiab] OR nicotine[tiab] OR tobacco[tiab] OR smoking[tiab])

AND ("use disorder*"[tiab] OR dependenc*[tiab] OR addict*[tiab]

OR abuse[tiab] OR cessation[tiab])

)

NOT ("Cannabis Use Disorder"[Mesh] OR "cannabis use disorder"[tiab]

OR "marijuana abuse"[tiab])

AND ("1975/01/01"[Date - Publication] : "2025/06/30"[Date - Publication])

AND humans[Mesh]

NOT (animals[Mesh] NOT humans[Mesh])

NOT ("Review"[Publication Type] OR "Meta-Analysis"[Publication Type]

OR "Systematic Review"[Publication Type] OR review[ti]

OR "systematic review"[tiab] OR meta-analys*[tiab]

OR "Study Protocol"[Publication Type] OR "Clinical Trial Protocol"[Publication Type]

OR protocol[ti] OR "study protocol"[tiab] OR "trial protocol"[tiab]

OR editorial[Publication Type] OR letter[Publication Type]

OR comment[Publication Type])

**Embase Search Query**

*Platform: Embase via Ovid. Dates: 1975 to 2025. Results: 6,595.*

1. exp *cannabis/

2. exp *cannabinoid/

3. exp *tetrahydrocannabinol/

4. exp *cannabidiol/

5. exp *dronabinol/

6. exp *nabilone/

7. (cannabis or marijuana or cannabinoid* or thc or

delta-9-tetrahydrocannabinol or cannabidiol or cbd or

dronabinol or nabilone or nabiximols or sativex).ti,ab.

8. (treat* or therap* or adjunct* or substitution or

replacement or maintenance or detox* or "withdrawal

management" or craving or "relapse prevention").ti,ab.

9. 7 and 8

10. 1 or 2 or 3 or 4 or 5 or 6 or 9

11. (rimonabant or SR141716 or taranabant or ibipinabant or

surinabant or otenabant or "CB1 antagonist" or "CB1

receptor antagonist" or "cannabinoid-1 antagonist" or

"inverse agonist*").ti,ab.

12. 10 not 11

13. exp *substance abuse/

14. exp *opiate addiction/

15. exp *alcohol dependence/

16. exp *cocaine dependence/

17. exp *amphetamine dependence/

18. exp *tobacco dependence/

19. (opioid* or heroin or cocaine or crack or amphetamine*

or methamphetamine or stimulant* or alcohol* or nicotine

or tobacco or smoking).ti,ab.

20. ("use disorder" or "use disorders" or dependenc* or

addict* or abuse or cessation).ti,ab.

21. 19 and 20

22. 13 or 14 or 15 or 16 or 17 or 18 or 21

23. exp cannabis addiction/

24. ("cannabis use disorder" or "marijuana abuse" or

"cannabis dependence" or "cannabis addiction").ti,ab.

25. 23 or 24

26. 12 and 22

27. 26 not 25

28. limit 27 to yr="1975-2025"

29. exp human/

30. exp animal/ not exp human/

31. 28 and 29

32. 31 not 30

33. (review or systematic review or meta analysis).pt.

34. review.ti.

35. (systematic review or meta-analys*).ti,ab.

36. (study protocol or trial protocol).ti,ab.

37. protocol.ti.

38. (editorial or letter or note).pt.

39. 33 or 34 or 35 or 36 or 37 or 38

40. 32 not 39

**eTable 1.** Synthesis Without Meta-analysis (SWiM) Reporting Checklist

*Reference: Campbell M, McKenzie JE, Sowden A, et al. Synthesis without meta-analysis (SWiM) in systematic reviews: reporting guideline. BMJ. 2020;368:l6890.*

| **Item** | **Description** | **Reported in manuscript** |
| --- | --- | --- |
| 1 | Grouping studies for synthesis | Methods, Data analysis: Studies grouped by SUD (OUD, AUD, CoUD, TUD, MUD) and endpoint (retention, relapse, craving, withdrawal severity, abstinence, consumption). No post-protocol changes to groupings. |
| 2 | Standardised metric and transformation methods | Methods, Data analysis: Direction-of-effect classification (Beneficial, No Significant Effect, Mixed/Partial, Harmful/Inferior) applied uniformly. No transformation of effect estimates; heterogeneity precluded a common effect size. |
| 3 | Synthesis methods | Methods, Data analysis: Structured narrative synthesis with direction-of-effect vote counting supplemented by tier-weighted framework (RCT 1.00 to qualitative 0.25). Meta-analysis precluded by design heterogeneity. |
| 4 | Criteria to prioritise results | Methods, Data analysis: Studies prioritised by design quality via prespecified tier weights. Direction-of-effect determined by original statistical significance thresholds. Cannabis exposure required to be isolated. |
| 5 | Investigation of heterogeneity | Methods and Results: Heterogeneity examined through stratification by SUD and endpoint, comparison of tier-weighted vs unweighted counts, disaggregation by design tier, and examination of outcome verification method. |
| 6 | Certainty of evidence | Methods: Risk of bias assessed using RoB-2 (RCTs), ROBINS-I (cohort studies), JBI checklists (cross-sectional, case series, qualitative). Tier-weighted framework provides design-aware calibration. |
| 7 | Data presentation methods | Results, Tables 1 and 2, Figures 2 to 4: Table 1 presents study characteristics ordered by design type. Table 2 cross-tabulates SUD endpoints by direction-of-effect with instance counts and tier score totals. Figure 2 displays direction-of-effect counts (Panel A) and tier-weighted totals (Panel B) by SUD. Figure 3 displays tier-weighted endpoint scores grouped by direction-of-effect category. Figure 4 displays the proportion of Beneficial findings and the proportion derived from lower-tier designs for each endpoint. |
| 8 | Reporting results | Results: 195 endpoint instances described by SUD, endpoint, and direction-of-effect with tier-weighted totals. Risk of bias profile summarized (12 of 17 RCTs low risk; 11 of 47 cohort studies low risk on ROBINS-I; 21 of 33 JBI-assessed studies low risk). Individual study contributions referenced throughout. Certainty calibrated via tier weights and outcome verification analysis showing inverse relationship between endpoint horizon and evidence tier (Figure 4). |
| 9 | Limitations of the synthesis | Discussion, Limitations: Heterogeneity, self-report reliance, residual confounding, insufficient RCT power, plausible publication bias, uneven representativeness. Tier weights involve subjective calibration. |

**eTable 2.** Studies Excluded at Full-Text Assessment

| **Study** | **Reason For Exclusion** |
| --- | --- |
| Belgrave et al., 1979 | Mechanistic or cognitive measures with no clinical SUD endpoint; Engaged healthy volunteers not SUD patients |
| Consroe et al., 1979 | Mechanistic or cognitive measures with no clinical SUD endpoint; Engaged healthy volunteers not SUD patients |
| Bird et al., 1980 | Mechanistic or cognitive measures with no clinical SUD endpoint; Engaged healthy volunteers not SUD patients |
| Joe et al., 1998 | Mechanistic or cognitive measures with no clinical SUD endpoint; Examined motivation as a predictor for treatment retention |
| Davstad et al., 2007 | No SUD endpoint recorded for analysis |
| Soyka et al., 2008 | Use of CB1 antagonists or inverse agonists (Rimonabant) |
| Lindsay et al., 2009 | No SUD endpoint recorded for analysis; Compared clinical characteristics between population cohorts |
| Rigotti et al., 2009 | Use of CB1 antagonists or inverse agonists (Rimonabant) |
| Morrison et al., 2010 | Use of CB1 antagonists or inverse agonists (Taranabant) |
| Cahill & Ussher 2011 | Use of CB1 antagonists or inverse agonists (Rimonabant and Taranabant) |
| Tonstad & Aubin 2012 | Use of CB1 antagonists or inverse agonists (Surinabant) |
| Evren et al., 2014 | Cannabis not isolated and not analysed as a distinct exposure against SUD treatment endpoints |
| White et al., 2014 | Cannabis not isolated and not analysed as a distinct exposure against SUD treatment endpoints |
| Jicha et al., 2015 | No SUD endpoint recorded for analysis; Concerned exclusively with safety outcomes |
| Butelman et al., 2017 | Mechanistic or cognitive measures with no clinical SUD endpoint |
| Heikman et al., 2017 | Cannabis not isolated and not analysed as a distinct exposure against SUD treatment endpoints; Polydrug exposure |
| Gilmore et al., 2018 | No SUD endpoint recorded for analysis; Mortality risk set as primary endpoint |
| Hindocha et al., 2018b | Mechanistic or cognitive measures with no clinical SUD endpoint; Reports only on impulsivity, delay discounting, and memory |
| Jarvis et al., 2018 | Cannabis not isolated and not analysed as a distinct exposure against SUD treatment endpoints |
| Robinson et al., 2018 | Use of CB1 antagonists or inverse agonists (Rimonabant) |
| Mongeau-Pérusse et al., 2022 | Mechanistic or cognitive measures with no clinical SUD endpoint; Focused on anxiety symptoms (BAI, VAS) and cortisol levels as a biomarker using data of parent trial |
| Roser et al., 2022 | Mechanistic or cognitive measures with no clinical SUD endpoint; Examined CBD use for management of sleep disorders, pain and anxiety |
| Rizkallah et al., 2022 | Mechanistic or cognitive measures with no clinical SUD endpoint; Focused on cognitive functioning outcomes using data from parent trial |
| Mok et al., 2023 | No SUD endpoint recorded for analysis; Evaluates correlates and predictors of cannabis use not efficacy or impact on SUD endpoints |
| Hall et al., 2024 | Mechanistic or cognitive measures with no clinical SUD endpoint; Engaged healthy volunteers not SUD patients |
| Herbet et al., 2025 | No SUD endpoint recorded for analysis; Reporting on laboratory biomarker AEA from parent trial |
| Hurzeler et al., 2025c | Mechanistic or cognitive measures with no clinical SUD endpoint; Investigated neurometabolites (GABA+, NAA, Glx) in the dorsal anterior cingulate cortex using 1H-MRS |

**eTable 3.** Study-level effect estimates and confidence intervals, by disorder and endpoint

| **Study** | **Endpoint** | **Direction** | **Effect Estimate** | **95% CI** |
| --- | --- | --- | --- | --- |
| **Opioid use disorder (OUD)** | | | | |
| Saxon et al., 1993 (29) | Retention | No significant effect | Cannabis status did not affect retention (NS) | NR |
| Saxon et al., 1996 (31) | Retention | No significant effect | Cox regression Exp(B)=1.08 (NS) | 0.97–1.20 |
| Budney et al., 1998 (32) | Retention | No significant effect | 65% vs 60% of weeks retained | NR |
| Church et al., 2001 (36) | Retention | No significant effect | Retention across groups F=1.932, p=0.159 | NR |
| Epstein & Preston, 2003 (38) | Retention | No significant effect | Retention not associated (p=.62–.79) | NR |
| Weizman et al., 2004 (39) | Retention | No significant effect | Time to leaving B=−0.17, p=0.21 | NR |
| Peles et al., 2006 (42) | Retention | No significant effect | 1-year retention 81.8% vs 74.1%, p=0.3 | NR |
| Nava et al., 2007 (43) | Retention | No significant effect | Retention NS (Kaplan–Meier) | NR |
| Schiff et al., 2007 (45) | Retention | Beneficial | OR 1.43 for 100% retention, p<.001 | 1.15–1.78 |
| Raby et al., 2009 (47) | Retention | Beneficial | Intermittent use HR 0.23 for dropout, p=0.001 | 0.09–0.57 |
| Chaudhry et al., 2012 (54) | Retention | No significant effect | Univariate p=0.04; multivariate NS | NR |
| Hill et al., 2013 (57) | Retention | No significant effect | Completion 48%/61%/56%, p=0.38 | NR |
| Potter et al., 2013 (60) | Retention | No significant effect | Retention/time-to-dropout NS | NR |
| Scavone et al., 2013 (61) | Retention | No significant effect | No impact on attendance/stabilization | NR |
| Hser et al., 2014 (62) | Retention | Harmful/Inferior | HR 2.10 for dropout, p<0.01 | 1.62–2.71 |
| Matson et al., 2014 (64) | Retention | Harmful/Inferior | THC+ urine HR 1.73 for not returning, p<0.001 | 1.14–2.63 |
| Abrahamsson et al., 2016 (66) | Retention | No significant effect | Univariate p=0.06; multivariate p=0.27 | NR |
| Bisaga et al., 2015 (67) | Retention | No significant effect | Retention 35% vs 35% at 8 weeks | NR |
| Levine et al., 2015 (71) | Retention | Harmful/Inferior | Dropout: males OR 5.00 (95% CI 1.61–14.29); females OR 9.09 | 2.33–33.33 (female); 1.61–14.29 (male) |
| Dayal et al., 2016 (74) | Retention | No significant effect | Bivariate z=2.06, p=0.03; multivariate OR 0.46 (p=0.49), OR 0.10 (p=0.17), NS | NR |
| Håkansson et al., 2016 (75) | Retention | No significant effect | 9-month retention NS (p=0.297; p=0.965) | NR |
| Dayal & Balhara, 2017 (78) | Retention | No significant effect | Past-month use NS in multivariate | NR |
| Franklyn et al., 2017 (79) | Retention | Harmful/Inferior | aHR 1.39 for dropout; heavy use aHR 1.48 | 1.06–1.83; 1.13–1.93 |
| Klimas et al., 2018 (85) | Retention | No significant effect | Discontinuation unadjusted HR 0.84, p=0.23; NS adjusted | 0.64–1.11 |
| Peles et al., 2018 (86) | Retention | No significant effect | Cannabis+ urine NS for retention (p=0.8) | NR |
| Eastwood et al., 2019 (87) | Retention | Mixed/Partial | Trajectory-dependent: AOR 2.39 (1.29–4.40) or AOR 0.50 (0.28–0.92) | 1.29–4.40; 0.28–0.92 |
| Lake et al., 2024 (115) | Retention | No significant effect | Discontinuation AOR 0.98 (0.66–1.45); >6 mo HR 0.91 (0.71–1.18) | 0.66–1.45; 0.71–1.18 |
| Wasserman et al., 1998 (33) | Relapse | Harmful/Inferior | Heroin lapse X²(1,N=74)=8.39, p<0.004; 6-mo X²=7.90, p<0.005 | NR |
| Epstein & Preston, 2003 (38) | Relapse | No significant effect | Heroin lapse HR ~1.20–1.54, p≥.095 | NR |
| Aharonovich et al., 2005 (40) | Relapse | No significant effect | HR 2.18 (NS) | 0.22–2.54 |
| Roux et al., 2011 (53) | Relapse | Harmful/Inferior | Daily cannabis associated with more opioid use (adjusted coefficient 0.28) | 0.08–0.47 |
| Lucas et al., 2013 (58) | Relapse | Beneficial | Survey (as above) | NR |
| Lions et al., 2014 (63) | Relapse | No significant effect | Univariate OR 2.81; NS in multivariate | 1.22–6.48 (univariate) |
| Lau et al., 2015 (70) | Relapse | Beneficial | Qualitative (as above) | NR |
| Proctor et al., 2016 (77) | Relapse | Harmful/Inferior | aOR 2.03 (opioid+ at 6 mo); aOR 5.19 (relapse at 12 mo) | 1.03–3.98; 1.26–21.47 |
| Zielinski et al., 2017 (82) | Relapse | Mixed/Partial | Overall OR 1.16; women OR 1.82 (1.18–2.82); men OR 1.11 (0.73–1.69) | 0.77–1.75 (overall) |
| Bunting et al., 2022 (104) | Relapse | No significant effect | Relapse by week 24 NS (p>0.05) | NR |
| Naji et al., 2022 (105) | Relapse | No significant effect | Time to relapse adjusted HR 1.03, p=0.84 | 0.78–1.36 |
| Shulman et al., 2025 (122) | Relapse | No significant effect | NS | NR |
| Saxon et al., 1993 (29) | Abstinence | No significant effect | Other-drug use THC+ median 6.5% vs THC− 6.3% (z=−0.48, NS) | NR |
| Budney et al., 1998 (32) | Abstinence | No significant effect | 8.4 vs 8.5 weeks abstinent | NR |
| Church et al., 2001 (36) | Abstinence | Mixed/Partial | Opiate+ urines 15% vs 60% vs 71.4%; F=9.381, p<0.001 (linear NS) | NR |
| Epstein & Preston, 2003 (38) | Abstinence | No significant effect | Opiate+ urines r²<.03 (NS) | NR |
| Alessi et al., 2011 (50) | Abstinence | No significant effect | Longest abstinence p=.95 | NR |
| Roux et al., 2011 (53) | Abstinence | Harmful/Inferior | Non-medical opioid use adjusted coefficient 0.28, p=0.01 | 0.08–0.47 |
| Somers & O’Connor, 2012 (56) | Abstinence | No significant effect | Adjusted OR 0.32 (unadjusted OR 0.84, 95% CI 0.57–1.25) | 0.07–1.57 |
| Hill et al., 2013 (57) | Abstinence | No significant effect | Opioid use NS (p>0.05) | NR |
| Potter et al., 2013 (60) | Abstinence | Beneficial | aOR 0.48 for abstinence at 24 weeks | 0.25–0.92 |
| Scavone et al., 2013 (61) | Abstinence | No significant effect | Opiate+ urines r=0.104, p=0.332 | NR |
| Abrahamsson et al., 2016 (66) | Abstinence | No significant effect | Opiate− vs + 5.9 vs 8.6 days (NS) | NR |
| Bisaga et al., 2015 (67) | Abstinence | No significant effect | Induction 66% vs 55% | NR |
| Lau et al., 2015 (70) | Abstinence | Beneficial | Qualitative (as above) | NR |
| Levine et al., 2015 (71) | Abstinence | No significant effect | Did not predict long-term abstinence | NR |
| Bagra et al., 2018 (83) | Abstinence | No significant effect | Opioid use 17.1% vs 13.8%, p=0.66 | NR |
| Lucas et al., 2019 (89) | Abstinence | Beneficial | Survey: opioids 59.3% abstinence, 18.4% ≥75% reduction | NR |
| Shams et al., 2019 (91) | Abstinence | No significant effect | Self-report aOR 0.45 (p=0.016); urine tox aOR 1.37 (NS) | 0.24–0.86 (self-report); 0.97–1.93 (urine) |
| Rosic et al., 2021 (102) | Abstinence | Mixed/Partial | Past-month use OR 1.03 (p=0.703); daily use OR 0.60 (0.46–0.78) | 0.87–1.23 (past-month); 0.46–0.78 (daily) |
| Shaw & Marcu, 2021 (103) | Abstinence | Beneficial | Case report: transition to naltrexone, no relapse | NR |
| Shulman et al., 2025 (122) | Abstinence | No significant effect | Cross-lagged: cannabis did not predict opioid use | NR |
| Nava et al., 2007 (43) | Craving | No significant effect | Craving fell in both groups (NS between) | NR |
| Lucas et al., 2013 (58) | Craving | Beneficial | Survey: 75.5% substituted cannabis; 67.7% cited less withdrawal | NR |
| Bisaga et al., 2015 (67) | Craving | No significant effect | No separate craving estimate reported | NR |
| Lau et al., 2015 (70) | Craving | Beneficial | Qualitative: substitute reducing cravings/withdrawal, maintaining abstinence | NR |
| Lofwall et al., 2016 (76) | Craving | Beneficial | Dronabinol 20 mg reduced desire for opiates | NR |
| Bagra et al., 2018 (83) | Craving | No significant effect | Craving 22.9% vs 16.9%, p=0.65 | NR |
| Hurd et al., 2019 (88) | Craving | Beneficial | Cue-induced craving (VAS-C) F=5.74, p=0.0047 | NR |
| Valleriani et al., 2020 (94) | Craving | Beneficial | Qualitative: 74% used cannabis to reduce other drug use | NR |
| Rosic et al., 2021 (102) | Craving | No significant effect | 6.9% said it helped craving | NR |
| Shaw & Marcu, 2021 (103) | Craving | Beneficial | Case report | NR |
| Bunting et al., 2022 (104) | Craving | No significant effect | Craving NS (p>0.05) | NR |
| Suzuki et al., 2022 (106) | Craving | Beneficial | Cue-induced craving 3.2→0.4, p=0.0046 | NR |
| Elkrief et al., 2023 (108) | Craving | No significant effect | Craving β=−0.05, p=0.49 | NR |
| Reddon et al., 2023 (111) | Craving | Beneficial | 44.4% used cannabis for cravings; aOR 2.13 | 1.07–4.27 |
| Suzuki et al., 2023 (112) | Craving | Beneficial | Post-cue craving 0.9 vs 2.4 (p=0.0046); difference 0.2 vs 1.3 (p=0.040) | NR |
| Bekier et al., 2024 (113) | Craving | Beneficial | 68.7% used cannabis to cope with craving (survey) | NR |
| Kudrich et al., 2024 (114) | Craving | Beneficial | Survey | NR |
| Wolkowicz et al., 2024 (117) | Craving | Mixed/Partial | Attentional bias NS overall; high-dose methadone subgroup bidirectional (p=.002) | NR |
| Gossop et al., 1991 (28) | Withdrawal severity | Mixed/Partial | Descriptive: of 22 cannabis users, 12 reported worse withdrawal, 6 reduced distress | NR |
| Hermann et al., 2005 (41) | Withdrawal severity | Mixed/Partial | Efficacy rated 3.6±1.0 on 5-point scale; 15% no effect, 37.5% worse | NR |
| Nava et al., 2007 (43) | Withdrawal severity | No significant effect | Withdrawal fell in both groups (NS between) | NR |
| Lucas et al., 2013 (58) | Withdrawal severity | Beneficial | Survey (as above) | NR |
| Scavone et al., 2013 (61) | Withdrawal severity | Beneficial | Lower withdrawal in users, p=0.006 | NR |
| Bisaga et al., 2015 (67) | Withdrawal severity | Beneficial | SOWS reduced vs placebo, p=0.006 | NR |
| Epstein & Preston, 2015 (68) | Withdrawal severity | No significant effect | Withdrawal F[1,104]=0.33, p=0.57; lagged r=0.01, p=0.69 | NR |
| Mayet et al., 2015 (72) | Withdrawal severity | No significant effect | OOWS NS (non-daily p=0.80; daily p=0.67) | NR |
| Lofwall et al., 2016 (76) | Withdrawal severity | Beneficial | Dronabinol 20–30 mg suppressed withdrawal (4.0–4.2 vs 6.7) | NR |
| Bagra et al., 2018 (83) | Withdrawal severity | No significant effect | Withdrawal 22.9% vs 13.8%, p=0.75 | NR |
| Bergeria et al., 2020 (92) | Withdrawal severity | Beneficial | SOWS 16.2 vs 27.8 (p<.05); VAS 35.3 vs 64.5 (p<.05) | NR |
| Valleriani et al., 2020 (94) | Withdrawal severity | Beneficial | Qualitative (as above) | NR |
| Mok et al., 2021 (100) | Withdrawal severity | Beneficial | Survey: 22% used cannabis for harm reduction (treating withdrawal 15%) | NR |
| Rosic et al., 2021 (102) | Withdrawal severity | No significant effect | 8.3% said it helped withdrawal; 74.9% no impact | NR |
| Shaw & Marcu, 2021 (103) | Withdrawal severity | Beneficial | Case report: COWS 14→3 by Day 7 | NR |
| De Aquino et al., 2023 (107) | Withdrawal severity | No significant effect | Subjective withdrawal NS vs placebo | NR |
| Elkrief et al., 2023 (108) | Withdrawal severity | No significant effect | Withdrawal β=0.09, p=0.36 | NR |
| Suzuki et al., 2023 (112) | Withdrawal severity | No significant effect | Withdrawal (COWS) NS | NR |
| Kudrich et al., 2024 (114) | Withdrawal severity | Beneficial | 41.9% used CBD for withdrawal; 79.7% agreed it helped (survey) | NR |
| Nirenberg et al., 1996 (30) | Consumption | No significant effect | Opiate+ urines 18% vs 11%; F(1,68)=0.90, p=.35 | NR |
| Saxon et al., 1996 (31) | Consumption | No significant effect | Illicit opioid use Beta=0.05 (NS) | NR |
| Best et al., 1999 (34) | Consumption | Beneficial | Heroin days 0.8 vs 1.6 vs 5.8; F=11.07, p<0.0001 | NR |
| Weizman et al., 2004 (39) | Consumption | No significant effect | Heroin use at 1 year (ANOVA, NS) | NR |
| Nava et al., 2007 (43) | Consumption | No significant effect | Opioid+ urines fell in both groups (NS between) | NR |
| Lucas et al., 2019 (89) | Consumption | Beneficial | Survey (as above) | NR |
| Valleriani et al., 2020 (94) | Consumption | Beneficial | Qualitative (as above) | NR |
| Mok et al., 2021 (100) | Consumption | Beneficial | Survey: substituting opioids 31% | NR |
| Elkrief et al., 2023 (108) | Consumption | No significant effect | Opioid use days β=−0.06, p=0.15 | NR |
| Lake et al., 2023 (110) | Consumption | Beneficial | Daily cannabis buffered low-dose risk (AOR 1.30 vs 1.86; interaction p=0.010) | NR |
| Reddon et al., 2023 (111) | Consumption | Beneficial | Reduced opioid use: daily aOR 3.87; mod–severe pain aOR 4.44 | 1.16–12.88; 1.52–12.97 |
| Bekier et al., 2024 (113) | Consumption | Beneficial | 44.8% substituted for heroin (survey) | NR |
| **Alcohol use disorder (AUD)** | | | | |
| Aharonovich et al., 2005 (40) | Relapse | Harmful/Inferior | HR 5.09, p<0.05 | 1.44–15.72 |
| Lucas et al., 2013 (58) | Relapse | Beneficial | Survey (as above) | NR |
| Lau et al., 2015 (70) | Relapse | Beneficial | Qualitative (as above) | NR |
| Alessi et al., 2011 (50) | Abstinence | No significant effect | Longest abstinence p=.95 | NR |
| Lau et al., 2015 (70) | Abstinence | Beneficial | Qualitative (as above) | NR |
| Lucas et al., 2019 (89) | Abstinence | Beneficial | Survey: alcohol 30.9% abstinence, 36.7% ≥75% reduction | NR |
| Lucas et al., 2013 (58) | Craving | Beneficial | Survey (as above) | NR |
| Lau et al., 2015 (70) | Craving | Beneficial | Qualitative (as above) | NR |
| Karoly et al., 2023 (109) | Craving | No significant effect | Craving (AUQ) NS (Bayesian CIs crossed zero) | NR |
| Hurzeler et al., 2025a (118) | Craving | Mixed/Partial | Craving reduced in recovery period (p=0.025); during cue NS (p=0.437) | NR |
| Hurzeler et al., 2025b (119) | Craving | No significant effect | Craving NS (AUQ p=0.40/0.49; VAS p=0.728) | NR |
| Kirkland et al., 2025 (120) | Craving | No significant effect | Craving (AUQ) NS | NR |
| Mueller et al., 2025 (121) | Craving | Beneficial | fsCBD reduced craving (PACS) p=0.014 (wk8), p<0.001 (wk16) | NR |
| Zimmermann et al., 2025 (123) | Craving | Beneficial | Blunted craving rise (p=0.025); fMRI cue craving (p=0.015) | NR |
| Reiman, 2007 (44) | Withdrawal severity | Beneficial | 43% reported fewer withdrawal symptoms (survey) | NR |
| Reiman, 2009 (48) | Withdrawal severity | Beneficial | 34% cited less withdrawal potential (survey) | NR |
| Lucas et al., 2013 (58) | Withdrawal severity | Beneficial | Survey (as above) | NR |
| Mok et al., 2021 (100) | Withdrawal severity | Beneficial | Survey (as above) | NR |
| Reiman, 2007 (44) | Consumption | Beneficial | 50% substituting cannabis for alcohol (survey) | NR |
| Reiman, 2009 (48) | Consumption | Beneficial | 40% reported substituting for alcohol (survey) | NR |
| Lucas et al., 2019 (89) | Consumption | Beneficial | Survey (as above) | NR |
| Karoly et al., 2020 (93) | Consumption | Mixed/Partial | Medical vs non-medical 2.04 vs 2.57 drinking days (p=.024) | NR |
| Karoly et al., 2021 (96) | Consumption | Beneficial | Fewer drinks/day β=0.180 (p=0.031); fewer drinking days β=0.141 (p=0.035) | NR |
| Mok et al., 2021 (100) | Consumption | Beneficial | Survey (as above) | NR |
| Kirkland et al., 2025 (120) | Consumption | No significant effect | Drinks over 7 days NS (p=0.261) | NR |
| Mueller et al., 2025 (121) | Consumption | No significant effect | Drinks/drinking day NS | NR |
| **Cocaine use disorder (CoUD)** | | | | |
| Alessi et al., 2011 (50) | Retention | Mixed/Partial | Retention 3.7 vs 5.6 weeks; interaction F(1,383)=4.2, p=.04 | NR |
| Oliveira et al., 2019 (90) | Retention | No significant effect | Retention NS (p>0.1) | NR |
| Dreher, 2002 (37) | Relapse | Beneficial | Qualitative | NR |
| Aharonovich et al., 2005 (40) | Relapse | No significant effect | HR 5.57 (NS) | 0.97–8.94 |
| Lucas et al., 2013 (58) | Relapse | Beneficial | Survey (as above) | NR |
| Viola et al., 2014 (65) | Relapse | Harmful/Inferior | Rehospitalization Beta=0.28, p=0.011 | NR |
| Gonçalves & Nappo, 2015 (69) | Relapse | Beneficial | Qualitative | NR |
| Lau et al., 2015 (70) | Relapse | Beneficial | Qualitative (as above) | NR |
| Oliveira et al., 2019 (90) | Relapse | No significant effect | Relapse NS (p>0.1) | NR |
| Mongeau-Pérusse et al., 2021 (101) | Relapse | No significant effect | Time to relapse NS (P=0.51) | NR |
| Labigalini et al., 1999 (35) | Abstinence | Beneficial | Case series: 68% ceased crack (mean 5.2 weeks) | NR |
| Dreher, 2002 (37) | Abstinence | Beneficial | 13/14 (92.8%) attributed cessation to cannabis | NR |
| Epstein & Preston, 2003 (38) | Abstinence | No significant effect | Cocaine+ urines r²<.03 (NS) | NR |
| Alessi et al., 2011 (50) | Abstinence | No significant effect | % negative samples p=.81 | NR |
| Green et al., 2012 (55) | Abstinence | Mixed/Partial | Per cannabis day: −5.4% (levodopa) vs +4.9% (placebo) | NR |
| Lau et al., 2015 (70) | Abstinence | Beneficial | Qualitative (as above) | NR |
| Meyer et al., 2021 (99) | Abstinence | Beneficial | Case report: complete abstinence, urine-verified to 12 weeks | NR |
| Labigalini et al., 1999 (35) | Craving | Beneficial | Reported reduced craving (descriptive) | NR |
| Dreher, 2002 (37) | Craving | Beneficial | Qualitative: reduced urge to use crack | NR |
| Li et al., 2005 (21) | Craving | No significant effect | Group×condition F=0.200, p=0.656 | NR |
| Oliveira & Nappo, 2008 (46) | Craving | Beneficial | Qualitative: cannabis as palliative, reduced craving | NR |
| Ribeiro et al., 2010 (49) | Craving | Mixed/Partial | Qualitative: reduced craving but binge-ritual risk | NR |
| Andrade et al., 2011 (51) | Craving | Beneficial | Qualitative: pitilho decreased craving | NR |
| Chaves et al., 2011 (52) | Craving | Beneficial | Qualitative: alleviate craving | NR |
| Lucas et al., 2013 (58) | Craving | Beneficial | Survey (as above) | NR |
| Viola et al., 2014 (65) | Craving | Harmful/Inferior | Craving 5.25 vs 2.48, p=0.004 | NR |
| Gonçalves & Nappo, 2015 (69) | Craving | Beneficial | Qualitative: reduced craving, longer inter-dose interval | NR |
| Lau et al., 2015 (70) | Craving | Beneficial | Qualitative (as above) | NR |
| Teixeira et al., 2015 (73) | Craving | Beneficial | Qualitative: managed craving (fissure) | NR |
| Giasson-Gariépy et al., 2017 (80) | Craving | Harmful/Inferior | Cue craving magnitude NS; rising baseline craving (time×group F=3.3, p=0.023) | NR |
| Valleriani et al., 2020 (94) | Craving | Beneficial | Qualitative (as above) | NR |
| Meneses-Gaya et al., 2021 (98) | Craving | No significant effect | Craving NS (F{10,230}=0.489, p=0.897) | NR |
| Mongeau-Pérusse et al., 2021 (101) | Craving | No significant effect | Craving NS (P=0.069) | NR |
| Labigalini et al., 1999 (35) | Withdrawal severity | Beneficial | Reported reduced withdrawal (descriptive) | NR |
| Oliveira & Nappo, 2008 (46) | Withdrawal severity | Beneficial | Qualitative | NR |
| Ribeiro et al., 2010 (49) | Withdrawal severity | Beneficial | Qualitative: helped withdrawal/paranoia | NR |
| Lucas et al., 2013 (58) | Withdrawal severity | Beneficial | Survey (as above) | NR |
| Viola et al., 2014 (65) | Withdrawal severity | Harmful/Inferior | Higher withdrawal increase (46.7% vs 16%) | NR |
| Teixeira et al., 2015 (73) | Withdrawal severity | Beneficial | Qualitative: managed comedown | NR |
| Valleriani et al., 2020 (94) | Withdrawal severity | Beneficial | Qualitative (as above) | NR |
| Viola et al., 2020 (95) | Withdrawal severity | Harmful/Inferior | Higher withdrawal vs non-use (p=0.028, d=0.43) | NR |
| Meneses-Gaya et al., 2021 (98) | Withdrawal severity | No significant effect | No superiority on anxiety/depression/sleep | NR |
| Mok et al., 2021 (100) | Withdrawal severity | Beneficial | Survey (as above) | NR |
| Mongeau-Pérusse et al., 2021 (101) | Withdrawal severity | No significant effect | Withdrawal NS | NR |
| Sociás et al., 2017 (81) | Consumption | Beneficial | Reduced crack use AOR 1.89 | 1.01–3.45 |
| Valleriani et al., 2020 (94) | Consumption | Beneficial | Qualitative (as above) | NR |
| Liu et al., 2021 (97) | Consumption | Beneficial | Indirect effect −0.09 via use intensity | −0.19 to −0.001 |
| Mok et al., 2021 (100) | Consumption | Beneficial | Survey: substituting stimulants 50% | NR |
| Mongeau-Pérusse et al., 2021 (101) | Consumption | No significant effect | Cocaine use days NS (P=0.682) | NR |
| Reddon et al., 2024 (116) | Consumption | No significant effect | Cocaine/crack reductions NS aOR 0.33 | 0.04–2.86 |
| **Tobacco use disorder (TUD)** | | | | |
| Lucas et al., 2019 (89) | Abstinence | Beneficial | Survey: tobacco 50.7% abstinence | NR |
| Morgan et al., 2013 (59) | Craving | No significant effect | Craving NS (TCQ, VAS) | NR |
| Hindocha et al., 2018 (84) | Craving | No significant effect | Craving NS (Bayes factor 7.08) | NR |
| Hindocha et al., 2018 (84) | Withdrawal severity | No significant effect | Withdrawal NS (Bayes factor 6.95) | NR |
| Morgan et al., 2013 (59) | Consumption | Beneficial | ~40% fewer cigarettes vs placebo, p=0.002; interaction p=0.054 | NR |
| Lucas et al., 2019 (89) | Consumption | Beneficial | Survey (as above) | NR |
| **Methamphetamine use disorder (MUD)** | | | | |
| Valleriani et al., 2020 (94) | Craving | Beneficial | Qualitative (as above) | NR |
| Reddon et al., 2024 (116) | Craving | Beneficial | 45.1% used cannabis for stimulant cravings (descriptive) | NR |
| Valleriani et al., 2020 (94) | Withdrawal severity | Beneficial | Qualitative (as above) | NR |
| Valleriani et al., 2020 (94) | Consumption | Beneficial | Qualitative (as above) | NR |
| Reddon et al., 2024 (116) | Consumption | Beneficial | Methamphetamine reductions aOR 0.08 | 0.02–0.37 |

CI = confidence interval; NR = not reported; NS = not significant

Estimates are reported at the level of individual studies, exactly as stated in the source reports, and are not pooled. The effect measure varies across studies (adjusted odds ratio, hazard ratio, regression coefficient, mean difference, or symptom-scale change), and estimates of different types are not combined. NR denotes that the study did not report a quantitative effect estimate for the endpoint, as is the case for qualitative, case, and descriptive survey studies. Where a 95% confidence interval was not reported, NR appears in that column even when a point estimate or p value was given. Confidence intervals are 95%.

**eFigure 1.** Risk of Bias Assessments

**eFigure 1a.** RoB 2 Risk of Bias Analysis for Crossover RCTs: Plot


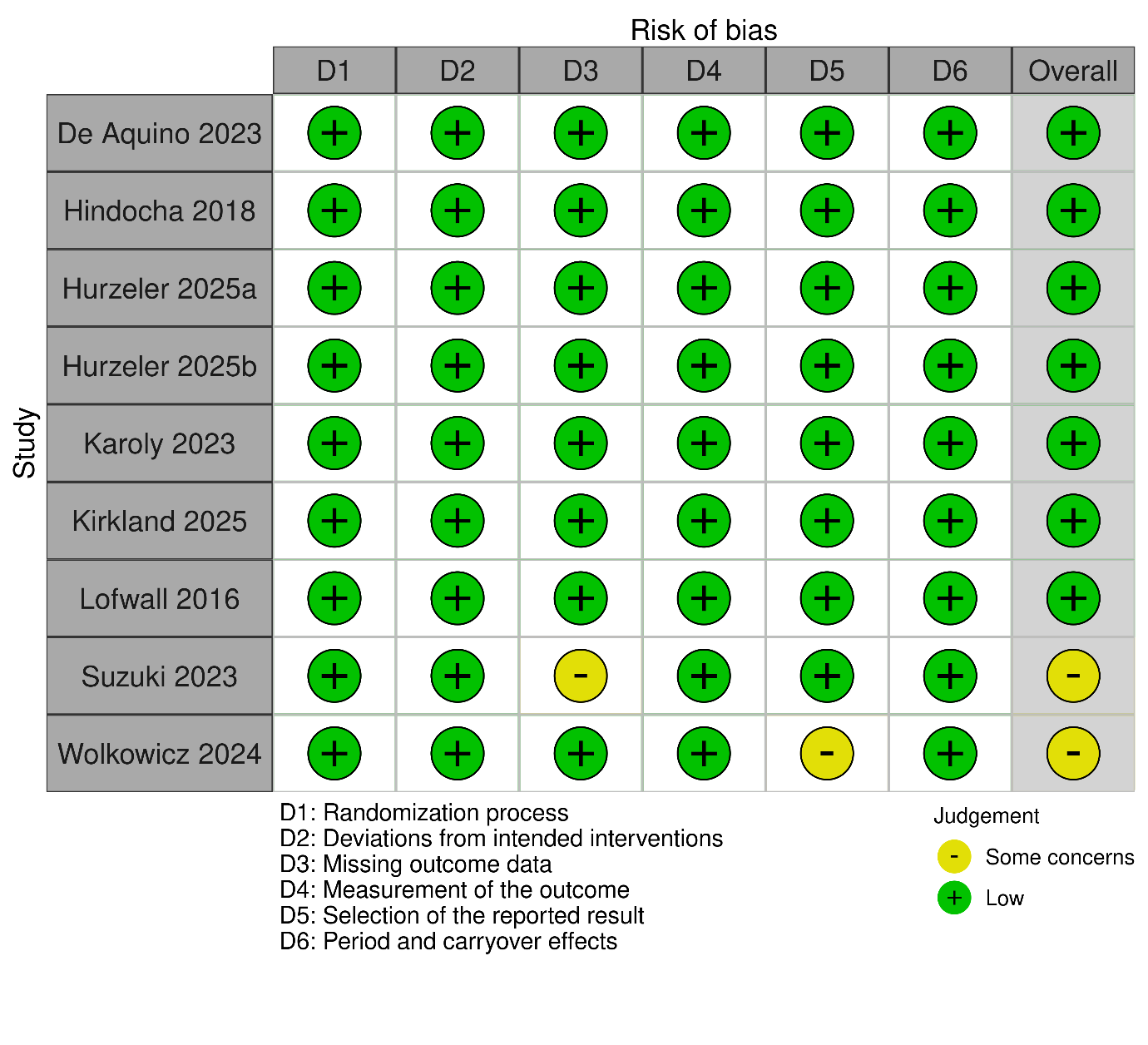


**eFigure 1b.** RoB 2 Risk of Bias Analysis for Crossover RCTs: Summary


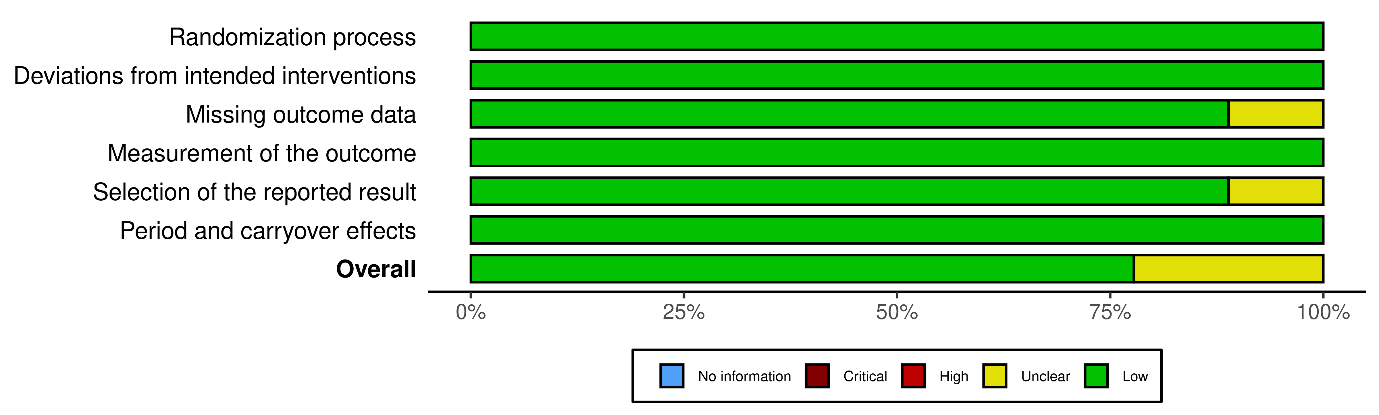


**eFigure 1c.** RoB 2 Risk of Bias Analysis for Parallel RCTs: Plot


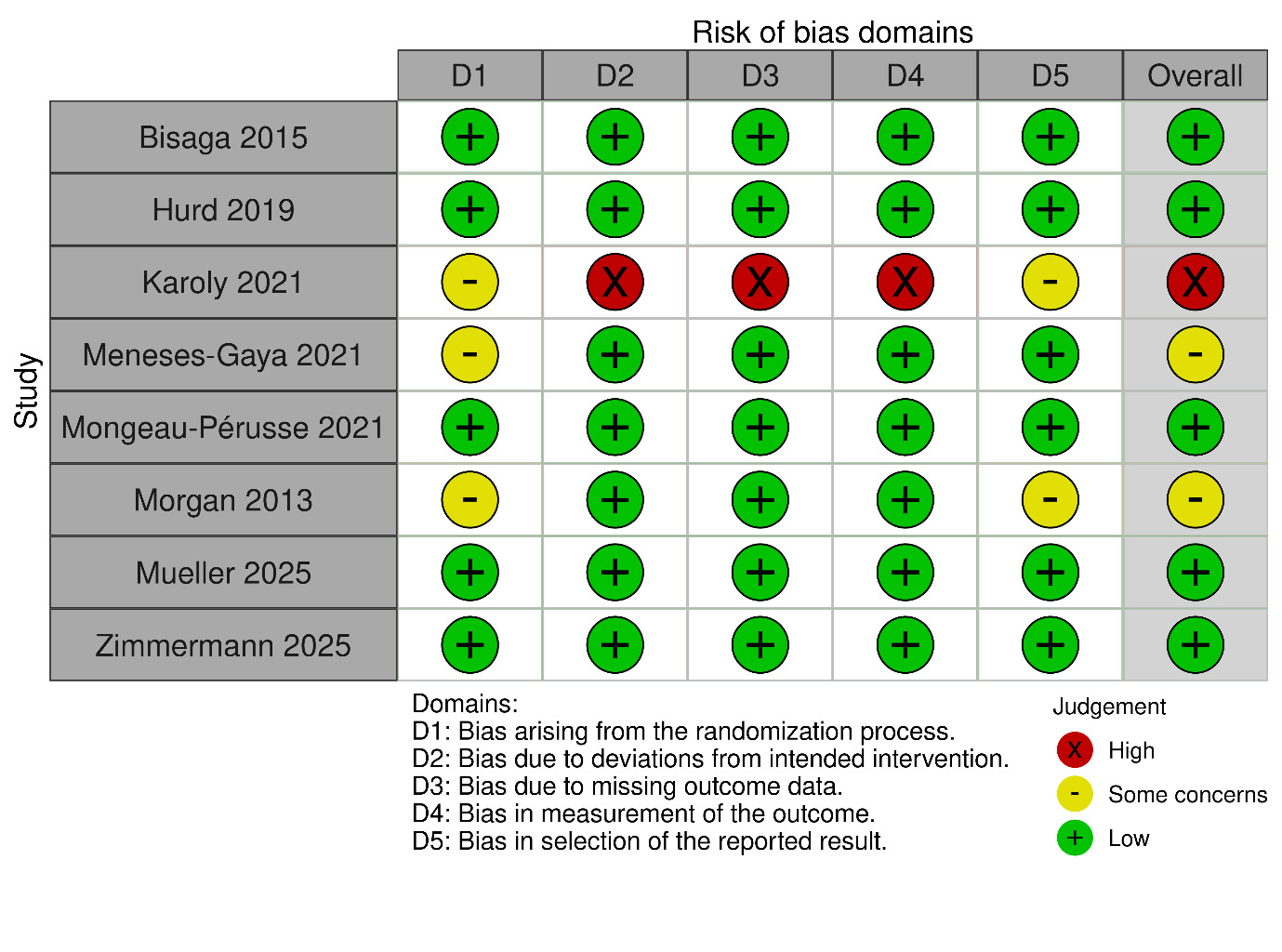


**eFigure 1d.** RoB 2 Risk of Bias Analysis for Parallel RCTs: Summary


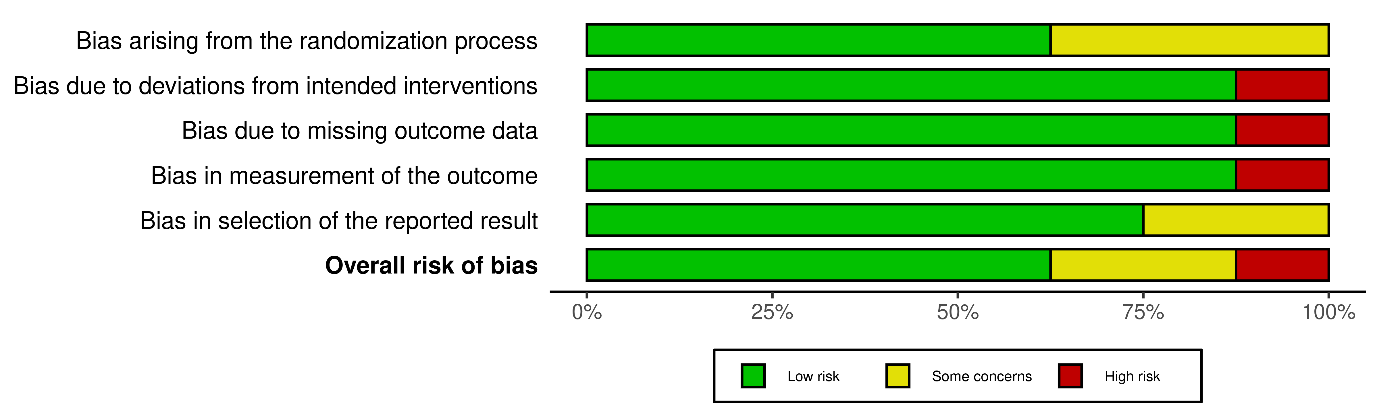


**eFigure 1e.** ROBINS-I Risk of Bias Analysis for Cohort Studies: Plot (part 1)


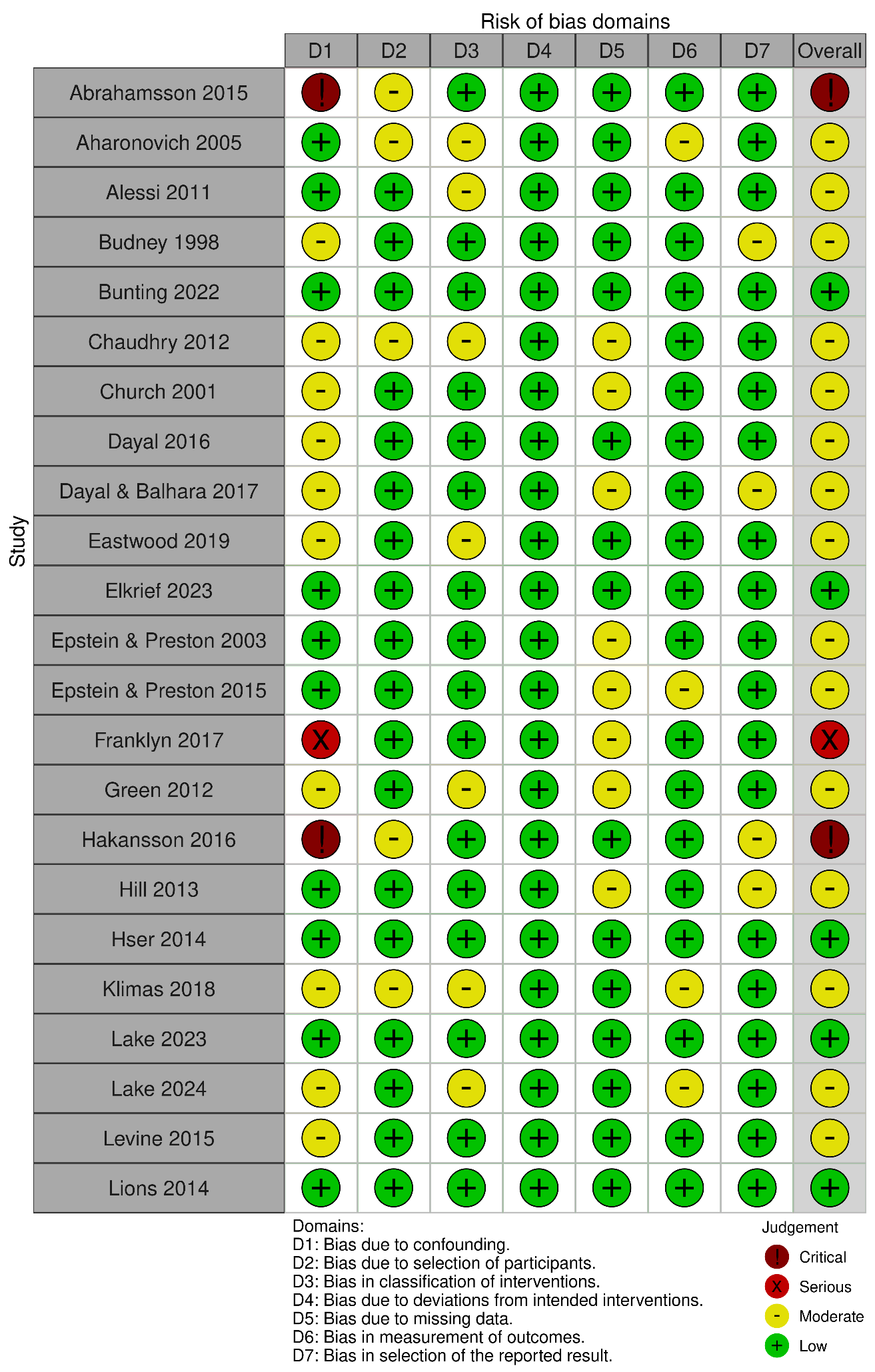


**eFigure 1f.** ROBINS-I Risk of Bias Analysis for Cohort Studies: Plot (part 2)


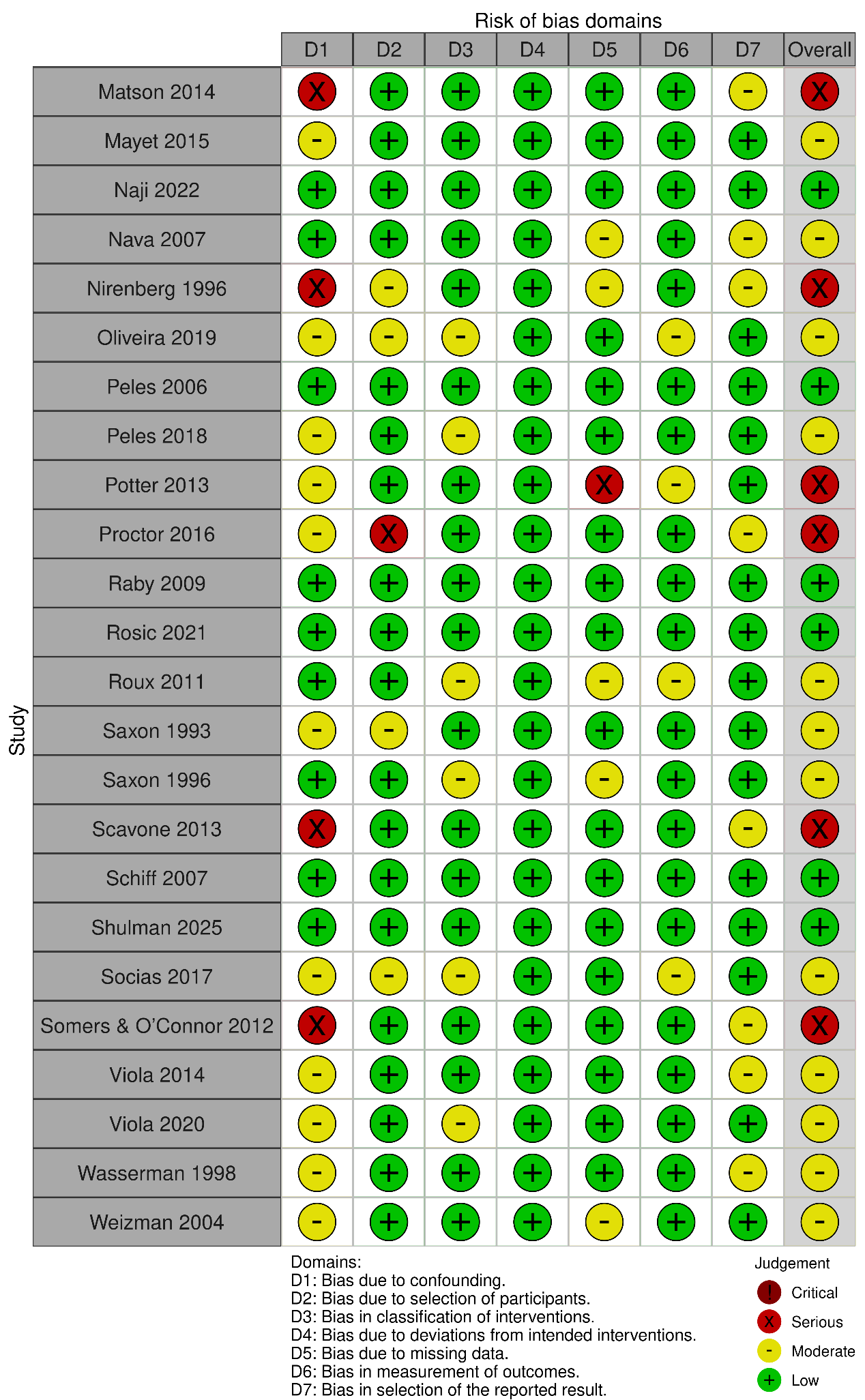


**eFigure 1g.** ROBINS-I Risk of Bias Analysis for Cohort Studies: Summary


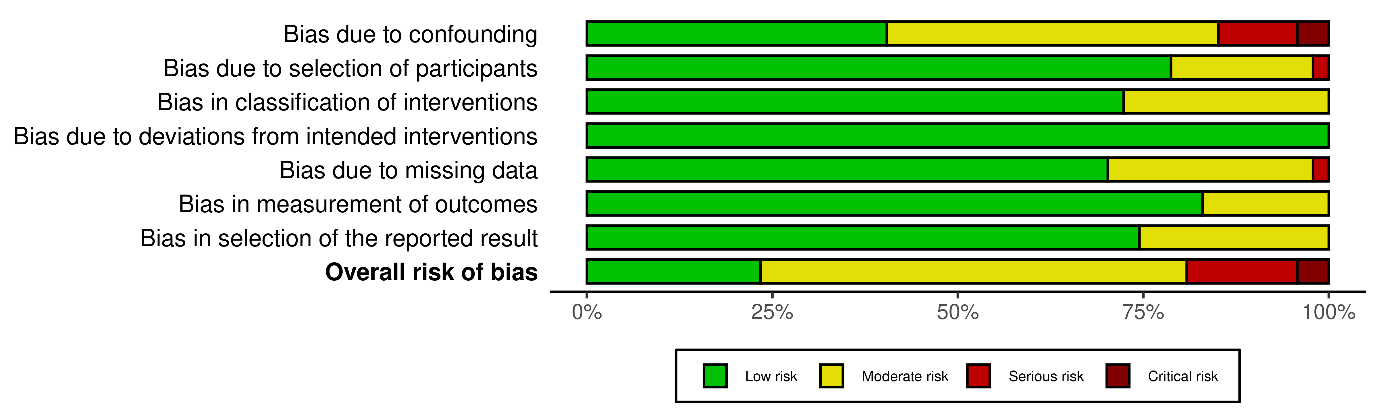


**eFigure 1h.** JBI Risk of Bias Analysis for Cross-Sectional, Case Series, Qualitative Studies: Table

**eFigure 1i.** Evidence Confidence By Disorder And Endpoint

| **Endpoint** | **Studies (n)** | **Design** | **Risk Of Bias** | **Predominant Direction** | **Confidence** | **Rating Basis** |
| --- | --- | --- | --- | --- | --- | --- |
| **Opioid use disorder (OUD)** | | | | | | |
| Retention | 27 | RCT and other designs | Low to moderate (5 low, 16 moderate, 6 serious) | No significant effect (20/27) | **High** | randomised evidence available; risk of bias low to moderate; consistent direction |
| Relapse | 12 | Cohort (multiple) | Low to moderate (6 low, 5 moderate, 1 serious) | No significant effect (6/12) | **Moderate** | multiple cohort studies; risk of bias low to moderate; direction not consistent across stronger designs |
| Abstinence | 20 | RCT and other designs | Low to moderate (6 low, 10 moderate, 4 serious) | No significant effect (13/20) | **High** | randomised evidence available; risk of bias low to moderate; consistent direction |
| Craving | 18 | RCT and other designs | Low to moderate (12 low, 6 moderate) | Beneficial (11/18) | **Moderate** | randomised evidence available; risk of bias low to moderate; direction not consistent across stronger designs |
| Withdrawal severity | 19 | RCT and other designs | Low to moderate (9 low, 8 moderate, 2 serious) | Beneficial (9/19) | **Moderate** | randomised evidence available; risk of bias low to moderate; direction not consistent across stronger designs |
| Consumption | 12 | Cohort (multiple) | Low to moderate (5 low, 6 moderate, 1 serious) | Beneficial (7/12) | **Moderate** | multiple cohort studies; risk of bias low to moderate; direction not consistent across stronger designs |
| **Alcohol use disorder (AUD)** | | | | | | |
| Relapse | 3 | Cohort (limited) | Low to moderate (1 low, 2 moderate) | Beneficial (2/3) | **Limited** | limited cohort evidence; risk of bias low to moderate; direction not consistent across stronger designs |
| Abstinence | 3 | Cohort (limited) | Low to moderate (1 low, 2 moderate) | Beneficial (2/3) | **Limited** | limited cohort evidence; risk of bias low to moderate; direction not consistent across stronger designs |
| Craving | 8 | RCT and other designs | Low to moderate (7 low, 1 moderate) | Beneficial (4/8) | **Moderate** | randomised evidence available; risk of bias low to moderate; direction not consistent across stronger designs |
| Withdrawal severity | 4 | Cross-sectional/case/qualitative | Low to moderate (1 low, 3 moderate) | Beneficial (4/4) | **Moderate** | lower-tier designs only; risk of bias low to moderate; consistent direction |
| Consumption | 8 | RCT and other designs | Low to moderate (3 low, 4 moderate, 1 serious) | Beneficial (5/8) | **Moderate** | randomised evidence available; risk of bias low to moderate; direction not consistent across stronger designs |
| **Cocaine use disorder (CoUD)** | | | | | | |
| Retention | 2 | Cohort (limited) | Low to moderate (0 low, 2 moderate) | Mixed/Partial (1/2) | **Limited** | limited cohort evidence; risk of bias low to moderate; direction not consistent across stronger designs |
| Relapse | 8 | RCT and other designs | Low to moderate (4 low, 4 moderate) | Beneficial (4/8) | **Moderate** | randomised evidence available; risk of bias low to moderate; direction not consistent across stronger designs |
| Abstinence | 7 | Cohort (multiple) | Low to moderate (3 low, 4 moderate) | Beneficial (4/7) | **Moderate** | multiple cohort studies; risk of bias low to moderate; direction not consistent across stronger designs |
| Craving | 16 | RCT and other designs | Low to moderate (12 low, 4 moderate) | Beneficial (10/16) | **Moderate** | randomised evidence available; risk of bias low to moderate; direction not consistent across stronger designs |
| Withdrawal severity | 11 | RCT and other designs | Low to moderate (6 low, 5 moderate) | Beneficial (7/11) | **Moderate** | randomised evidence available; risk of bias low to moderate; direction not consistent across stronger designs |
| Consumption | 6 | RCT and other designs | Low to moderate (5 low, 1 moderate) | Beneficial (4/6) | **Moderate** | randomised evidence available; risk of bias low to moderate; direction not consistent across stronger designs |
| **Tobacco use disorder (TUD)** | | | | | | |
| Abstinence | 1 | Cross-sectional/case/qualitative | Low to moderate (0 low, 1 moderate) | Beneficial (1/1) | **Very limited** | lower-tier designs only; risk of bias low to moderate; single study |
| Craving | 2 | RCT | Low to moderate (1 low, 1 moderate) | No significant effect (2/2) | **Limited** | randomised evidence available; risk of bias low to moderate; consistent direction |
| Withdrawal severity | 1 | RCT | Low to moderate (1 low, 0 moderate) | No significant effect (1/1) | **Limited** | randomised evidence available; risk of bias low to moderate; single study |
| Consumption | 2 | RCT and other designs | Low to moderate (0 low, 2 moderate) | Beneficial (2/2) | **Limited** | randomised evidence available; risk of bias low to moderate; direction not consistent across stronger designs |
| **Methamphetamine use disorder (MUD)** | | | | | | |
| Craving | 2 | Cross-sectional/case/qualitative | Low to moderate (2 low, 0 moderate) | Beneficial (2/2) | **Limited** | lower-tier designs only; risk of bias low to moderate; consistent direction |
| Withdrawal severity | 1 | Cross-sectional/case/qualitative | Low to moderate (1 low, 0 moderate) | Beneficial (1/1) | **Very limited** | lower-tier designs only; risk of bias low to moderate; single study |
| Consumption | 2 | Cross-sectional/case/qualitative | Low to moderate (2 low, 0 moderate) | Beneficial (2/2) | **Limited** | lower-tier designs only; risk of bias low to moderate; consistent direction |
